# Characterization of six canine prostate adenocarcinoma and three transitional cell carcinoma cell lines derived from primary tumor tissues as well as metastasis

**DOI:** 10.1371/journal.pone.0230272

**Published:** 2020-03-13

**Authors:** Eva-Maria Packeiser, Marion Hewicker-Trautwein, Heike Thiemeyer, Annika Mohr, Johannes Junginger, Jan Torben Schille, Hugo Murua Escobar, Ingo Nolte

**Affiliations:** 1 Small Animal Clinic, University of Veterinary Medicine Hannover, Hannover, Germany; 2 Department of Internal Medicine, Medical Clinic III, Clinic for Hematology, Oncology and Palliative Care, University Medical Centre Rostock, Rostock, Germany; 3 Department of Pathology, University of Veterinary Medicine Hannover, Hannover, Germany; Southern Illinois University School of Medicine, UNITED STATES

## Abstract

Canine prostate adenocarcinoma (PAC) and transitional cell carcinoma (TCC) of prostate and urinary bladder are highly invasive and metastatic tumors of closely neighbored organs. Cell lines are valuable tools to investigate tumor mechanisms and therapeutic approaches *in vitro*. PAC in dogs is infrequent, difficult to differentiate from TCC and usually characterized by poor prognosis, enhancing the value of the few available cell lines. However, as cell lines adapt to culturing conditions, a thorough characterization, ideally compared to original tissue, is indispensable. Herein, six canine PAC cell lines and three TCC cell lines were profiled by immunophenotype in comparison to respective original tumor tissues. Three of the six PAC cell lines were derived from primary tumor and metastases of the same patient. Further, two of the three TCC cell lines were derived from TCCs invading into or originating from the prostate. Cell biologic parameters as doubling times and chemoresistances to commonly used drugs in cancer treatment (doxorubicin, carboplatin and meloxicam) were assessed. All cell lines were immunohistochemically close to the respective original tissue. Compared to primary tumor cell lines, metastasis-derived cell lines were more chemoresistant to doxorubicin, but equally susceptive to carboplatin treatment. Two cell lines were multiresistant. COX-2 enzyme activity was demonstrated in all cell lines. However, meloxicam inhibited prostaglandin E2 production in only seven of nine cell lines and did neither influence metabolic activity, nor proliferation. The characterized nine cell lines represent excellent tools to investigate PAC as well as TCC in prostate and urinary bladder of the dog. Furthermore, the profiled paired cell lines from PAC primary tumor and metastasis provide the unique opportunity to investigate metastasis-associated changes PAC cells undergo in tumor progression. The combination of nine differently chemoresistant PAC and TCC cell lines resembles the heterogeneity of canine lower urinary tract cancer.

## Introduction

Cancer cell lines represent useful tools to investigate tumorigenesis and to establish new therapeutic approaches [1]. For research in rare but severe tumor entities like canine prostate cancer [2,3], cell lines are even more valuable, as access to tissue samples and primary cultures is limited. However, for reliable interpretation of *in vitro* studies, a thorough characterization of the used cell lines is inevitable.

In general, cell lines are established from tumor-burdened individuals. Ideally, investigated features of the primary tumor are representative for the tumor type or subtype and stay preserved in the derived cell line [4]. However, clonal selection and adaption to culturing conditions over multiple passages can affect features like gene expressions and sensitivities against chemotherapeutic acting drugs [4–6]. Accordingly, the matched characterization of cell lines and respective tissues of origin allows a comprehensive evaluation in which terms a cell line actually represents the tumor entity and can therefore be used as suitable model.

Canine prostate adenocarcinoma (PAC) and transitional cell carcinoma (TCC) both show highly invasive growth and metastatic behavior that limit treatment options [7,8]. Several cytostatic drugs and cyclooxygenase 2 (COX-2) inhibitors appear to prolong survival times in TCC patients [8,9], whereas few studies support slight effects of COX-2 inhibitors solely or combined with chemotherapeutic agents against canine PAC [10,11]. Comparable to human castrate-resistant prostate cancer, canine PAC is mostly androgen-independent and therefore refractory to androgen depriving or suppressing treatments [7,12]. Positive immunostaining of the tumor suppressor p53 in human PAC and TCC is mutation-related [13,14] and thus a negative prognostic factor. In canine osteosarcoma, mutations of *p53* have also been detected and correlated with poor survival [15]. Further, immunoreactivity for p53 was demonstrated in canine PAC and TCC [16–18], suggesting p53 as negative prognostic factor.

The majority of canine prostatic tumors are adenocarcinomas, arisen from glandular tissue and further classified by a Gleason-like scoring [19,20]. However, PAC is likely to infiltrate the closely neighbored bladder-neck and likewise, TCC also infiltrates prostatic tissue, or originates from the prostatic urethra or periurethral ducts [21–26]. Differentiation between PAC and TCC in prostatic tumors is recommended [20,26], as further research might discover differences in therapeutic options and prognosis. However, a clear distinction based on clinical imaging, cytology, histopathology and molecular markers is still challenging [9,27–30].

Metastasis is a major limiting factor for treatment and prognosis of cancer [31,32]. While popular human PAC cell lines like PC-3 and LNCaP were derived from metastasis, available canine cell lines were usually established from primary tumors [33–39]. Studies characterizing cellular features associated with naturally occurring PAC metastasis are limited, as there are no paired cell lines derived from both primary tumor and metastases of the same species, ideally from the same patient. PAC in dogs is comparable with castration-resistant prostate cancer in men [21,40,41] and canine TCC resembles human invasive bladder cancer [42–44]. Thus, therapeutic options developed for one species might likewise be beneficial for the other. While researchers in human medicine have access to numerous well-characterized PAC and TCC cell lines and sublines [1,45], only few specified canine cell lines are described [16,35,38,39,46,47].

The aim of this study was to equally profile a large set of cell lines derived from canine PAC, PAC metastasis, as well as TCC localized in urinary bladder and prostate. Therefore, the cell lines and their primary tissue counterparts were characterized with respect to growth characteristics, immunophenotype, cellular phenotype, sensitivity to chemotherapeutic drugs and COX-2 expression.

## Materials and methods

### Patients and tissue samples

Nine dogs with carcinomas derived from the prostate or urinary bladder that were presented to the Small Animal Clinic of the University of Veterinary Medicine Hannover between 2003 and 2015 were included in this study. Medical patient data are summarized in S1 Table. All dogs except from patient no. 7 were euthanized shortly after diagnosis because of advanced stage of the disease and poor prognosis. Patient no. 7 died from chemoresistant and metastatic tumor recurrence one year after surgery. Owners of all euthanized patients consented to leave their dogs’ bodies in the clinic for research purposes. Tissue samples were taken from neoplastic areas, as verified by histopathology, within two hours after euthanasia. From patient no. 4, additional tissue samples from two separate sublumbal lymph node metastases were obtained. Altered tissue samples of one patient no. 7 were obtained directly after medically indicated surgery from the resected tumor in agreement with the patient´s owner. Tissue samples of patient no. 5 were not available as the patient’s owner declined surgery or necropsy. Tissues were divided for routine histopathology and for immunohistochemistry (formalin-fixed paraffin-embedded, FFPE) and for further cell culture in Hank’s Medium (Hank’s salts, Biochrom GmbH, Berlin, Germany). Sections of FFPE samples were stained with hematoxylin and eosin (HE). According to national legislation, this study does not require ethics approvals, as samples were taken from animals euthanized for medical reasons or from material obtained for therapeutic or diagnostic purpose.

### Classification of original tissues

Sections of FFPE tissues (Table 1) were examined by a certified and experienced veterinary pathologist and classified as PAC, metastasis of PAC or TCC according to the guidelines proposed by Palmieri et al., Valli et al. and Knapp et al. [48–50]. Gleason grading was performed as described by Palmieri and Grieco [19]. Images were taken in 400x magnification using an Imager.Z1 microscope (Carl Zeiss AG, Oberkochen, Germany).

**Table 1:** Patients, tissue samples and cell lines.

Patient	Original tissue	Histological classification	Cell line	Former name
1	P1	PAC	TihoDPro**Adcarc1258**	CT1258
2	P2	PAC	TihoDPro**Adcarc0846**	DT08/46
3	P3	PAC	TihoDPro**Adcarc1508**	DT15/08
4	P4.1	PAC	TihoDPro**Adcarc1511.1**	none
	Ln4.2	PAC metastasis	TihoDPro**Metadcarc1511.2**	none
	Ln4.3	PAC metastasis	TihoDPro**Metadcarc1511.3**	none
5	n.a.	TCC*	TihoDProCarc/**TCC0840**	DT08/40
6	P6	TCC	TihoDPro**TCC1509**	DT15/09
7	B7	TCC	TihoDUrt**TCC1506**	DT15/06

Original tissue (P = Prostate, B = urinary bladder, Ln = lymph node); cell lines’ names are explained as institution (Tiho = University of Veterinary Medicine Hannover); species (D = dog); tissue origin (Pro = prostate; Urt = urinary tract (urinary bladder)); diagnosis (Adcarc = adenocarcinoma; Carc = carcinoma; Metadcarc = metastasis of an adenocarcinoma); abbreviations of cell lines written in bold; none = cell lines have not been published yet; n.a. = tissue of patient no. 5 is missing, as the patient owners declined surgery and necropsy;

*diagnosis by cytology of cells obtained by fine needle aspiration biopsy.

### Primary cultures and cell lines

Cases include cell lines CT1258 and DT08/40 [35,51], which were previously characterized by karyotype. CT1258 and DT08/40, as well as some other herein profiled cell lines have already been used in various studies [52–58] and were renamed consistently according to Table 1. TCC0840 has been established from cells obtained by fine needle aspiration [51], all other cell lines were established as described for Adcarc1258 [35]. Cells were cultivated in Medium 199 (Life Technologies GmbH, Darmstadt, Germany) containing 20% fetal calf serum (FBS Superior, Biochrom GmbH), 200 IU/ml penicillin and 200 mg/ml streptomycin (Biochrom GmbH). At passage 30, fetal calf serum was reduced to 10% and cell lines were characterized after another five passages of adaption.

Tissue samples from sclera, conjunctiva, subdermal tissue and oral mucosa were obtained from an eleven-year-old female neutered Galgo Espanol shortly after euthanasia with prior consent of the owner. The dog was euthanized due to decompensated heart failure, lameness and insolinoma. Fibroblast cultures were established as described for cell line Adcarc1258 [35] and cultured in DMEM / F12 (1:1) medium, stable Glutamine, 1.2 g / L NaHCO_2_ (PAN-biotech, Aidenbach, Germany) containing 10% fetal calf serum (FBS Superior, Biochrom GmbH), 200 IU/ml penicillin and 200 mg/ml streptomycin (Biochrom GmbH).

### Description of cell morphology in cell lines

FFPE embedded cell pellets were stained with HE. Images were taken at 400x magnification using an Imager.Z1 microscope (Carl Zeiss AG, Oberkochen, Germany).

### Comparative characterization of cellular origin, p53 and COX-2 expression in cell lines and tissue of origin

FFPE tissues and FFPE embedded cell pellets of three consecutive passages were immunohistochemically stained with mouse monoclonal antibodies raised against pan-cytokeratin (pan-CK, detecting CK1, CK5, CK10, CK14), CK7, CK8/18, uroplakin III (UPIII), vimentin, E-Cadherin (E-Cad) and Calponin (Calp) and p53, as well as goat polyclonal IgG detecting COX-2 (S2 Table). Cross reactivity with canine tissue was either declared by the manufacturers or based on the literature [59–62] as summarized in S2 Table. If not described otherwise, all incubation steps were performed at room temperature. For antigen retrieval, slides were cooked in citrate buffer by microwave for 20 minutes or digested by proteinase K for 40 minutes for the CK7 antibody. Nonspecific binding was blocked using inactivated normal goat serum and slides were incubated with primary antibodies for 75 minutes. CK8/18, Calp, p53 and COX-2 antibodies required incubation overnight at 4 °C. This was followed by incubation with goat anti-mouse secondary antibodies for 45 minutes for monoclonal primary antibodies and with horse anti-goat secondary antibodies for COX-2. Staining was rendered visible performing an indirect avidin-biotin-peroxidase staining procedure according to the manufacturer. For CK8/18, Calp and COX-2, staining was amplified by the use of 0.5% biotinylated tyramine and 0.06% H_2_O_2_ followed by a secondary incubation with the avidin-biotin complex. 3,3’-diaminobenzidine was used to generate a brown color reaction and sections were counterstained with hematoxylin. Canine non-neoplastic tissue or squamous cell carcinoma tissue with confirmed immunoreactivity served as positive controls. Those tissues were acquired from leftover samples sent to routine histopathology diagnostics with consent of the owners (S2 Table). Host- and isotype-matched antibodies were applied as negative controls instead of primary antibodies (S2 Table). Tissue samples from healthy dogs served as positive controls and host- and isotype-matched antibodies were applied as negative controls instead of primary antibodies (S2 Table). Staining intensities and percentages of COX-2 and p53 expressing cells in cell pellets and in neoplastic areas of tissue samples were scored as proposed by Sorenmo et al. [10] and Pagliarone et al. [18]. Following Pagliarone et al., samples with less than 10% randomly scattered cells expressing p53 were considered negative.

### Fibroblast exclusion

All tumor cell lines were seeded in a 96-well plate 7,500 cells/well. Canine fibroblast cells (each 5,000 cells/well) from sclera, conjunctiva, subdermal tissue and oral mucosa were used as positive control. All cells lines were stained with a rabbit anti collagen VI antibody 1:100 (ab6588, Abcam, Cambridge, UK) as described before [63] applying a goat polyclonal anti-rabbit IgG H+L secondary antibody (Alexa Fluor 647, Abcam) diluted 1:250. Nuclei were stained by 4,6-diamidino-2-phenylindole diluted 1:1000 (DAPI, Sigma-Aldrich Chemie GmbH, Munich, Germany). Fluorescence images were taken utilizing an inverse microscope DMI6000 B in 100x magnification (Leica Microsystems GmbH, Wetzlar, Germany).

### Determination of doubling times

Cell lines were seeded in 6-well plates 2*10^5^ cells/well. Medium was exchanged after 48 h. After 24, 48, 72 and 96 h of cultivation, three wells of each cell line were washed with phosphate buffered saline (PBS) for removal of dead cells. The cells were then detached with 1 ml TrypLE^™^Express (Life Technologies GmbH) and counted with an automated cell counter Cellometer Auto-T4 (Nexcelcom Bioscience, Lawrence, MA, USA). Exponential equations n(t) = n_0_*r^t^ of growth curves were established, with n representing the cell count after t [h], n_0_ the cell count after 24 h, t the time [h] and r the specific growth rate of each cell line. Doubling times (DT) [h] were calculated using the formula DT [h] = ln(2) / ln(r).

### Surveillance of growth behavior over time

Cell lines were seeded in 96-well plates 7,500 cells/well. Morphology and growth behavior were surveyed with a live cell Imaging microscope (DMI6000 B, Leica Microsystems, Wetzlar, Germany) over 72 h in 100x magnification. Pictures of the same position were taken every 15 minutes with the program LAS AF 2.6.0. A heating unit, a humidifier and regulated CO_2_ supply achieved constant cultivation conditions as described above.

### Chemotherapeutic agents

Doxorubicin (Doxo-Cell 2 mg/ml, STADApharm GmbH, Bad Vilbel, Germany) was diluted to 100 μM in PBS and carboplatin (Carbo-CELL 10 mg/ml, STADApharm GmbH) was diluted to 10 mM in cell culture medium. Therefore, solvent controls in cell culture medium used for controls in doxorubicin and carboplatin inhibition were not necessary. Meloxicam was purchased from Selleck Chemicals (Houston, USA) and solved in dimethyl sulfoxide (DMSO) to a concentration of 75 mM. Solutions were sterile filtered and aliquots were stored at -20 °C.

### Chemosensitivity to doxorubicin and carboplatin

The inhibitory effect of doxorubicin and carboplatin on metabolic activity was ascertained by MTS assay (CellTiter 96^®^ AQueous One Solution Cell Proliferation Assay, Promega, Mannheim, Germany). All cell lines were seeded in 96-well plates 7,500 cells/well. The next day, medium was exchanged, and cells were exposed in quadruplets to increasing concentrations of doxorubicin (1, 10, 50, 100, 200, 500, 700, 1000, 1500 and 2000 nM) or carboplatin (0.5, 1, 5, 10, 20, 50, 100, 200, 500 and 1000 μM). Dilutions were chosen based on doxorubicin serum concentrations in patient dogs [64] and on carboplatin plasma concentrations in healthy beagle dogs [65] after infusion of therapeutic dosages. Controls of each cell line were supplied with fresh medium. After 72 h of exposure, MTS assay CellTiter 96^®^ AQueous One Solution Cell Proliferation Assay, Promega) was conducted according to manufacturer’s protocol. The absorbance was recorded at 490 nM using a Synergy2 plate reader (BioTek, Bad Friedrichshall, Germany). Blanks were subtracted.

To determine the influence of doxorubicin and carboplatin on total cell numbers, cells were seeded in 6-well plates 1.5*10^5^ cells/well. The next day, cells were treated as described for MTS assay, though for carboplatin, 0.5 and 1000 μM used in MTS assay were omitted as they showed little differences to 0 and 2000 μM in MTS assays. After 72 h of exposure to doxorubicin or carboplatin, cells were counted as described for doubling times.

Results were normalized to the respective medium control. Dose-response curves were established for MTS and total cell numbers and inhibitory concentrations 50 (IC50) were calculated using GraphPad Prism Software 3.0 (GraphPad Soſtware Inc., San Diego, CA, USA).

### Induction of apoptosis by doxorubicin and carboplatin

Additionally, proportions of vital, apoptotic and late apoptotic cells were assessed after doxorubicin and carboplatin exposure. Cells were seeded in 6-well plates 1.5*10^5^ cells/well and exposed to the respective concentrations of doxorubicin or carboplatin used in MTS assay the next day. For carboplatin, 0.5 and 1000 μM were omitted again, as these concentrations showed little differences to 0 and 2000 μM in MTS assays. Controls were supplied with fresh medium. After 72 h of exposure, medium including floating cells and debris was collected. Adherent cells were detached with 1 ml TrypLE^™^Express (Life Technologies GmbH), pooled with the respective medium and centrifuged for 10 min at 160 g. The obtained pellets were stained with Annexin V-FITC Detection Kit plus (PromoCell, Heidelberg, Germany) according to manufacturer’s protocol and fluorescence signals were measured by flow cytometry using a MACSQuant^®^ Analyzer 10 (Miltenyi Biotec, Bergisch Gladbach, Germany). Data analysis was performed with FlowJo 10.3 software. Debris was excluded and proportions of vital, apoptotic and late apoptotic cells were determined.

### Inhibition of COX-2

For meloxicam response regarding total cell number and metabolic activity, cells were seeded in 24-well plates 4*10^4^ cells/well and in 96-well plates 7,500 cells/well. The next day, medium was exchanged and cells were exposed to 1μM, which resembles the therapeutic steady state plasma concentration in dogs [66], and 10 μM meloxicam in quadruplets. Controls were incubated with 0.13% (v/v) DMSO. After 72 h of exposure, the MTS assay procedure was conducted as described for doxorubicin and carboplatin inhibition and cells were counted as described for doubling times.

The functionality of COX-2 enzyme was ascertained by production of prostaglandin E2 (PGE2). Each cell line was seeded in a density of 7.5*10^4^ cells/well in 12-well plates. The next day, medium was changed and cells were treated in duplicates with 1 and 10 μM meloxicam. Controls were incubated with 0.13% (v/v) DMSO. After 24 h of exposure, medium was collected, centrifuged for 10 min at 160 g for removal of cells and debris and stored at -80 °C. PGE2 concentrations in medium samples were measured in duplicates using a competitive ELISA (Prostaglandin E2 ELISA monoclonal, Cayman Chemical, Ann Arbor, USA) according to manufacturer’s protocol.

### Statistics

Statistical analysis of data was performed with SAS software 7.1 (SAS Institute Inc., Cary, NC, USA). Means and standard deviations are displayed. For comparison to untreated controls in metabolic activity, cell count and proportions of vital, apoptotic and late apoptotic cells, Dunnett’s test for multiple comparisons was applied. Additionally, Student’s t-tests were performed for comparison between PGE2 concentrations at 1 and 10 μM meloxicam. The confidence levels were set to 95% (p < 0.05; *), 99% (p < 0.01; **) and 99.9% (p < 0.001; ***). All experiments were conducted three times independently, except from cell count and apoptosis rate of doxorubicin and carboplatin response, which were conducted four times and from PGE2 concentrations, which were measured once.

## Results

### Classification of original tissues

FFPE prostate tissue samples P1, P2, P3 and P4.1 were histopathologically classified as infiltrative PAC (Table 1). All four PAC had a single growth pattern which was either solid (patient no. 1 and 3) or acinar/ductal (patient no. 2 and 4) with Gleason scores of 10 (patient no. 1 and 3), 9 (patient no. 4) or 8 (patient no. 2). A full necropsy performed on patient no. 3 revealed the presence of metastases in lungs, brain and uvea. In patient no. 4, PAC metastasis was diagnosed in lymph node tissue samples Ln4.2 and Ln4.3. Cytological examination of cells obtained from patient no. 5 revealed carcinoma, most likely TCC. FFPE urinary bladder tissue sample B7 was confirmed as TCC. Prostate tissue P6 was diagnosed as TCC, larger tissue samples of the respective patient revealed TCC, invading into prostate tissue.

### Description of cell morphology in cell lines

**Fig 1:**
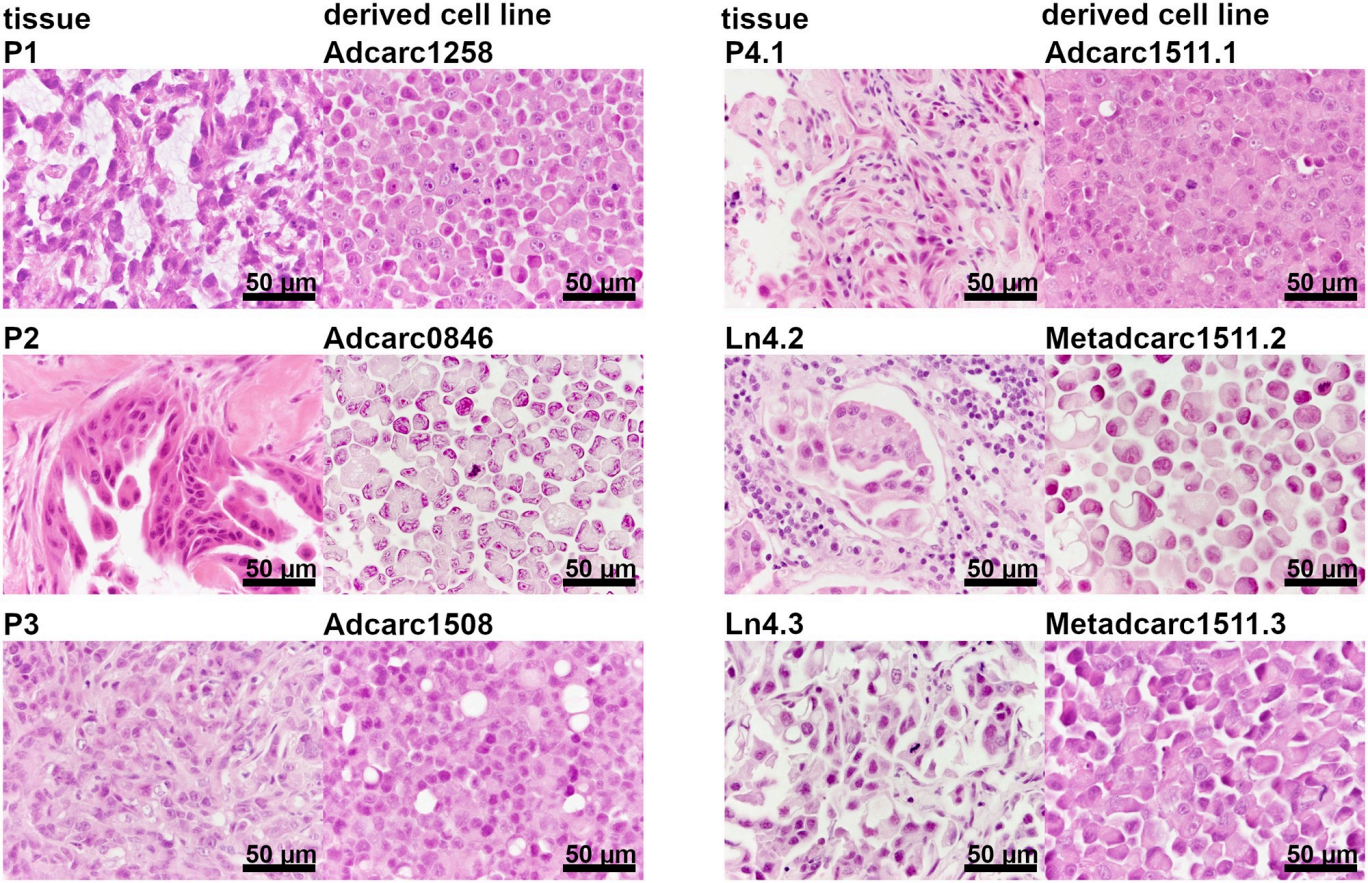
Formalin-fixed and HE-stained PAC tissue samples and cell pellets.

**Fig 2:**
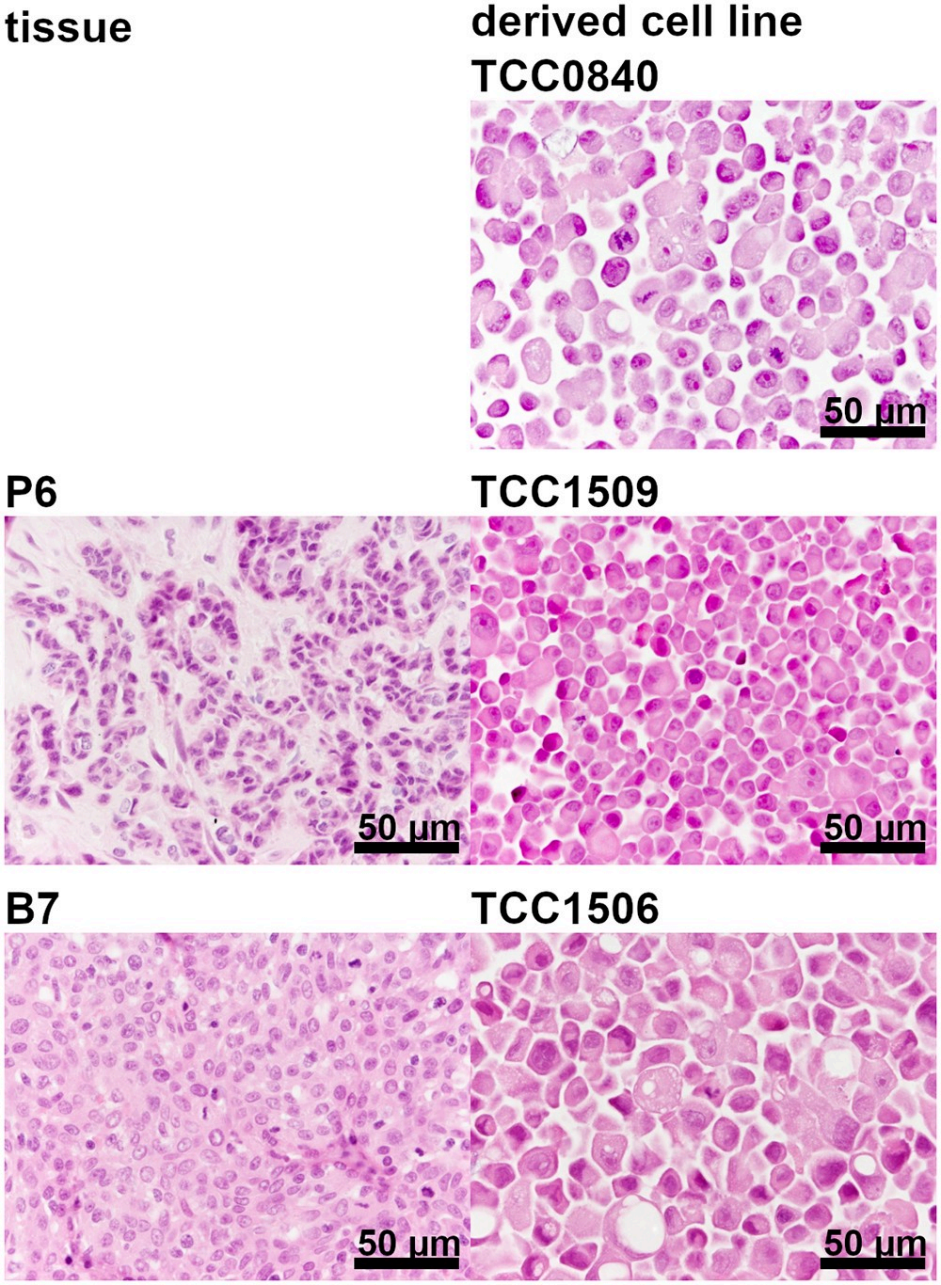
Formalin-fixed and HE-stained TCC tissue samples and cell pellets.

In both metastasis-derived cell lines Metadcarc1511.2 and Metadcarc1511.3, cells were distinctly larger than cells of the primary tumor cell line Adcarc1511.1 and showed numerous cytoplasmic vacuoles, which were also observed in Adcarc1508 and TCC0840 cells (Figs 1 and 2). Adcarc1508 cells were of heterogeneous size with predominant small cells and few large, multinucleated cells. Adcarc1258 and TCC1509 cells were smaller compared to the other cell lines.

### Comparative characterization of cellular origin, p53 and COX-2 expression in cell lines and tissue of origin

Tissue material of P2 was not sufficient for immunohistochemical staining. The overall immunohistochemical pattern for cellular origin, epithelial character and COX-2 expression remained stable in cell lines compared to original tissues, with few exceptions (Table 2). Immunophenotype changes were observed for vimentin (five gains), pan-CK (four losses), CK7 (three losses), CK8/18 and E-Cad (one loss each). Cell line Adcarc1258 and respective original tissue were negative for all tested cellular origin markers. UPIII-staining was negative in all samples, while Calp and COX-2 staining could be detected in all samples. Moderate nuclear immunoreactivity for p53 protein was observed in B7 and corresponding cell line TCC1506 with 40–70% and >70% positive cells respectively. COX-2 staining could be observed in all samples with index increases and decreases in intensity.

**Table 2:** Comparative immunohistochemical staining of cell lines and original tumor tissues.

Cell line and tissue	pan-CK	CK7	CK8/18	UPIII	vimentin	E-Cad	Calp	COX-2 index*	p53 index**
P1	-	-	-	-	+	+	+	2	-
Adcarc1258	-	-	-	-	+	-	+	12	-
Adcarc0846	+	+	+	-	-	+	+	8	-
P3	+	+	+	-	-	+	+	12	-
Adcarc1508	+	+	+	-	+	+	+	8	-
P4.1	+	+	+	-	-	+	+	8	-
Adcarc1511.1	-	-	+	-	+	+	+	12	-
Ln4.2	+	+	+	-	-	+	+	8	-
Metadcarc1511.2	-	+	+	-	+	+	+	12	-
Ln4.3	+	+	+	-	-	+	+	8	-
Metadcarc1511.3	-	+	+	-	+	+	+	12	-
TCC0840	+	+	+	-	-	+	+	12	-
P6	+	+	+	-	-	+	+	12	-
TCC1509	-	-	-	-	+	+	+	8	-
B7	+	+	+	-	-	+	+	12	3+; m
TCC1506	+	-	+	-	-	+	+	12	4+; m

* COX-2 staining was quantified according to Sorenmo et al. 2004;

** p53 staining was quantified according to Pagliarone et al. 2016; + = positive; − = negative; m = moderate; 3+ = 40–70% of cells stained positive; 4+ = <100% of cells stained positive. Tissue samples for Adcarc0846 and TCC0840 were not listed, as P2 tissue material was not sufficient for immunohistochemical staining and TCC0840 was established from fine needle aspiration biopsy and no tissue sample was available.

### Fibroblast exclusion

In immunofluorescence analysis, all fibroblast cells used as controls stained positive for collagen VI, whereas all tumor cell lines were negative (S1 Fig).

### Determination of doubling times

Doubling times of cell lines varied between 18.2 and 38.5 h (Table 3). The longest doubling times were recorded in both metastasis-derived cell lines. Adcarc1508 and TCC1509 reached confluence between 72 and 96 h of cultivation and therefore were limited in proliferation. Determined cell count values for these two cell lines after 96 h were excluded for calculations of doubling times. Ascertained curves fitted with coefficients of determination (R^2^) between 0.9151 and 0.9999.

**Table 3:** Doubling times (DT) of cell lines in h.

PAC	DT [h]	PAC-Metastasis	DT [h]	TCC	DT [h]
Adcarc1258	21.6	Metadcarc1511.2	37.9	TCC0840	28.6
Adcarc0846	26.2	Metadcarc1511.3	38.5	TCC1506	19.9
Adcarc1508	18.2			TCC1509	21.3
Adcarc1511.1	27.9				

### Surveillance of growth behavior over time

TCC0840, Adcarc0846, TCC1506, Adcarc1508, Adcarc1511.1, Metadcarc1511.2 and Metadcarc1511.3 grew in monolayers in cobblestones-like patterns. Adcarc0846 and Adcarc1508 tended to form piles when reaching confluence. Adcarc1258 and TCC1509 had low anchoring potential and did not establish uniform monolayers in culture. Instead, cells formed prominent protrusions when attached to the well bottom and tended to form aggregates with increasing cell density. Videos of growth behavior are provided as S1–S9 Videos.

### Chemosensitivity to doxorubicin and carboplatin

**Fig 3:**
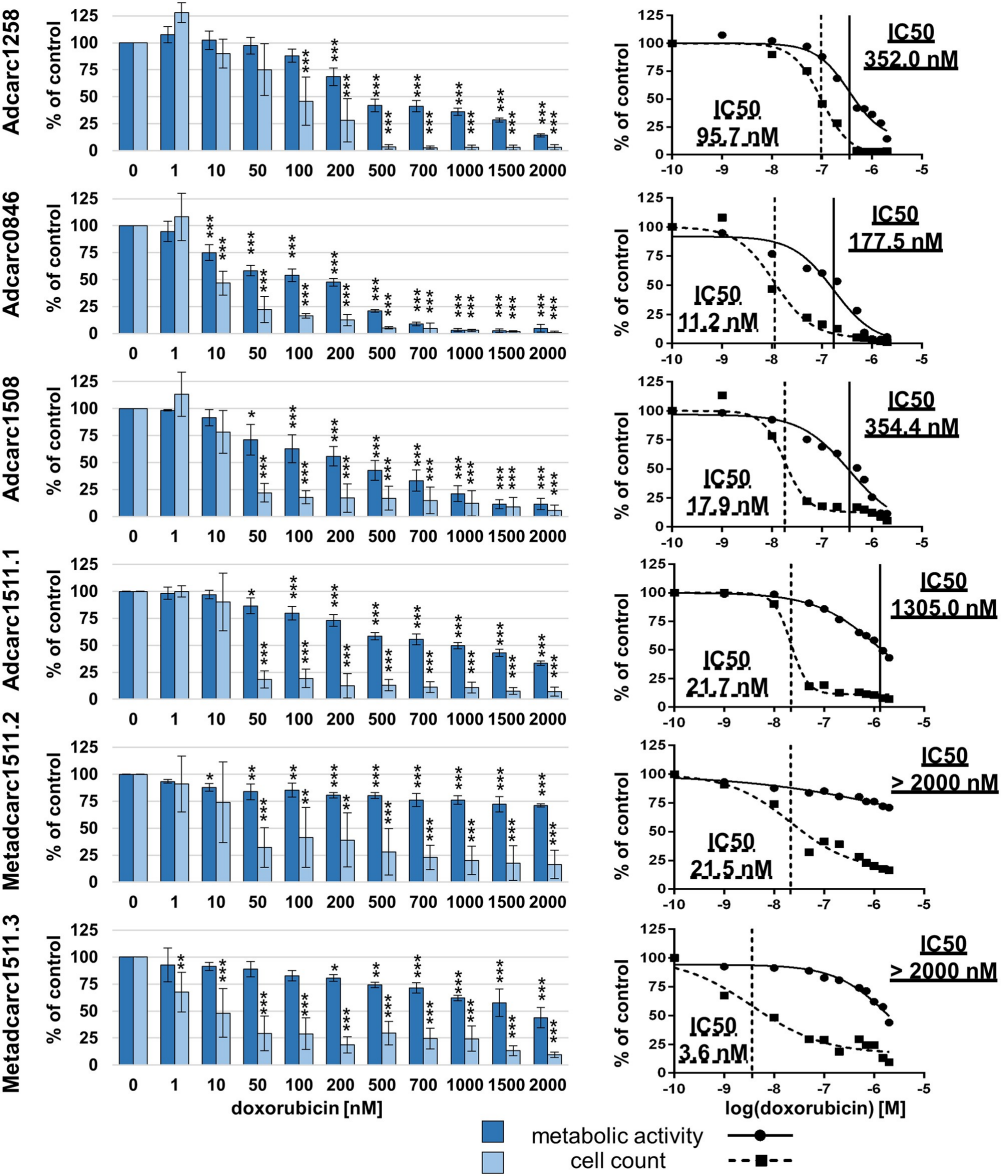
Metabolic activities and cell counts of PAC cell lines after 72 h incubation with doxorubicin. Means and standard deviation are displayed, n = 3 for metabolic activity, n = 4 for cell count, significances are referred to controls. *p < 0.05, p** < 0.01 and p*** <0.01.

**Fig 4:**
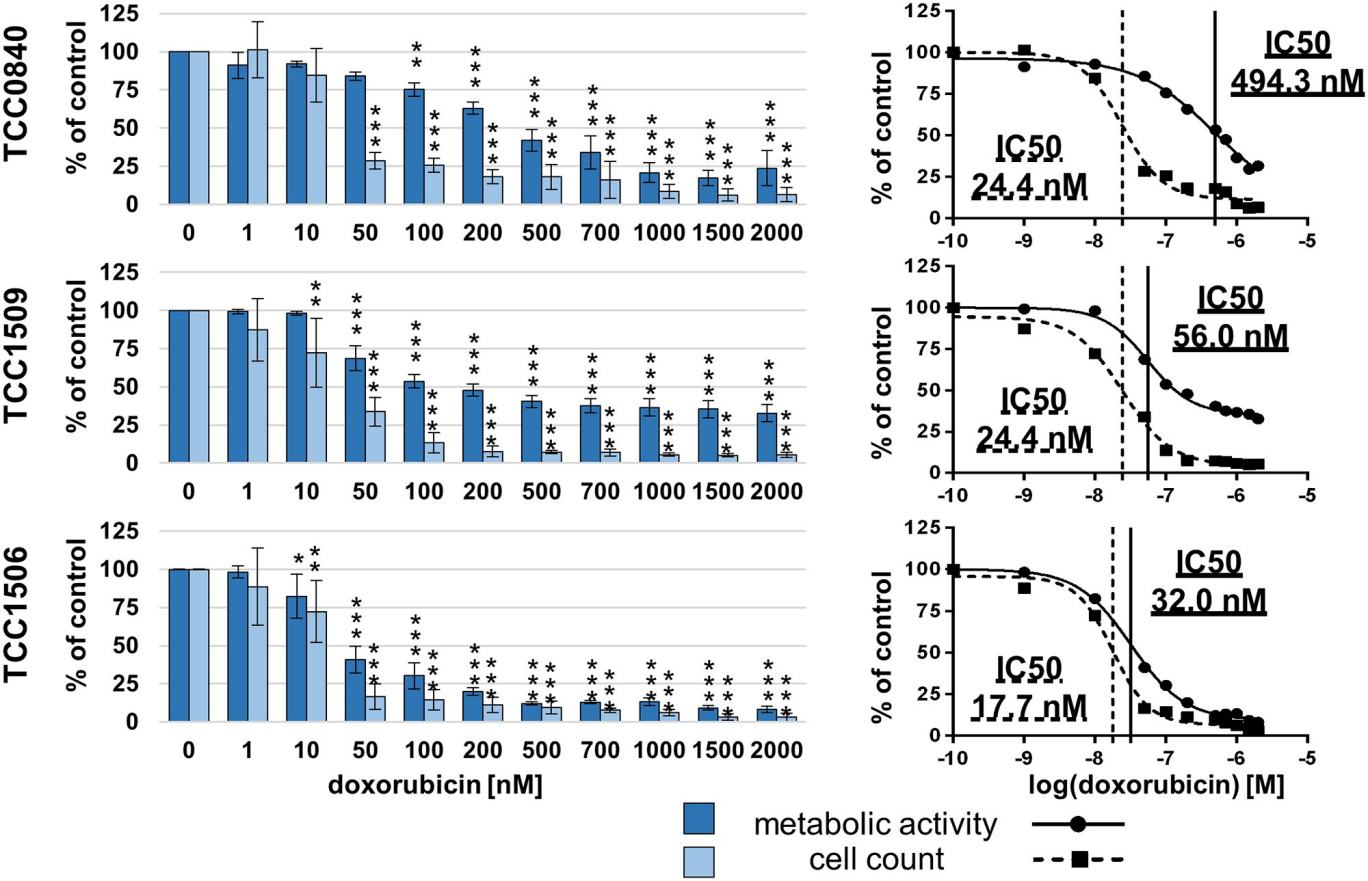
Metabolic activities and cell counts of TCC cell lines after 72 h incubation with doxorubicin. Means and standard deviation are displayed, n = 3 for metabolic activity, n = 4 for cell count, significances are referred to controls. *p < 0.05, p** < 0.01 and p*** <0.01.

After 72 h of exposure to doxorubicin, all cell lines showed decreased metabolic activity at 200 nM and higher (Figs 3 and 4). Reductions of metabolic activities below 33% could be achieved in all cell lines derived from primary tumors at 2000 nM. IC50 values of metabolic activities varied between 177.5 and 1305.0 nM for PAC primary tumors and between 32.0 and 494.3 nM for TCC (Table 4). Metabolic activity of both cell lines established from metastases could not be decreased to 50% after exposure to 2000 nM doxorubicin.

**Table 4:** IC50 values for doxorubicin and carboplatin after 72 h exposure, n = 3.

IC50 values	Doxorubicin [nM]		Carboplatin [μM]	
	Metabolic activity	Cell count	Metabolic activity	Cell count
Adcarc1258	352.0	85.0	97.7	6.6
Adcarc0846	177.5	14.8	106.0	4.3
Adcarc1508	354.4	14.3	67.7	8.5
Adcarc1511.1	1305.0	19.0	86.1	3.8
Metadcarc1511.2	> 2000.0	19.7	38.3	10.9
Metadcarc1511.3	> 2000.0	10.9	46.1	8.9
TCC0840	494.3	23.6	129.3	4.4
TCC1509	56.0	24.4	33.6	3.8
TCC1506	32.0	15.5	39.8	9.0

Cell count IC50 values for doxorubicin varied between 3.6 nM in Metadcarc1511.3 and 95.7 nM in Adcarc1258. At 2000 nM doxorubicin, total cell numbers in all PAC and TCC derived cell lines were reduced below seven percent, total cell numbers of cell lines originating from metastases were reduced below 16%.

**Fig 5:**
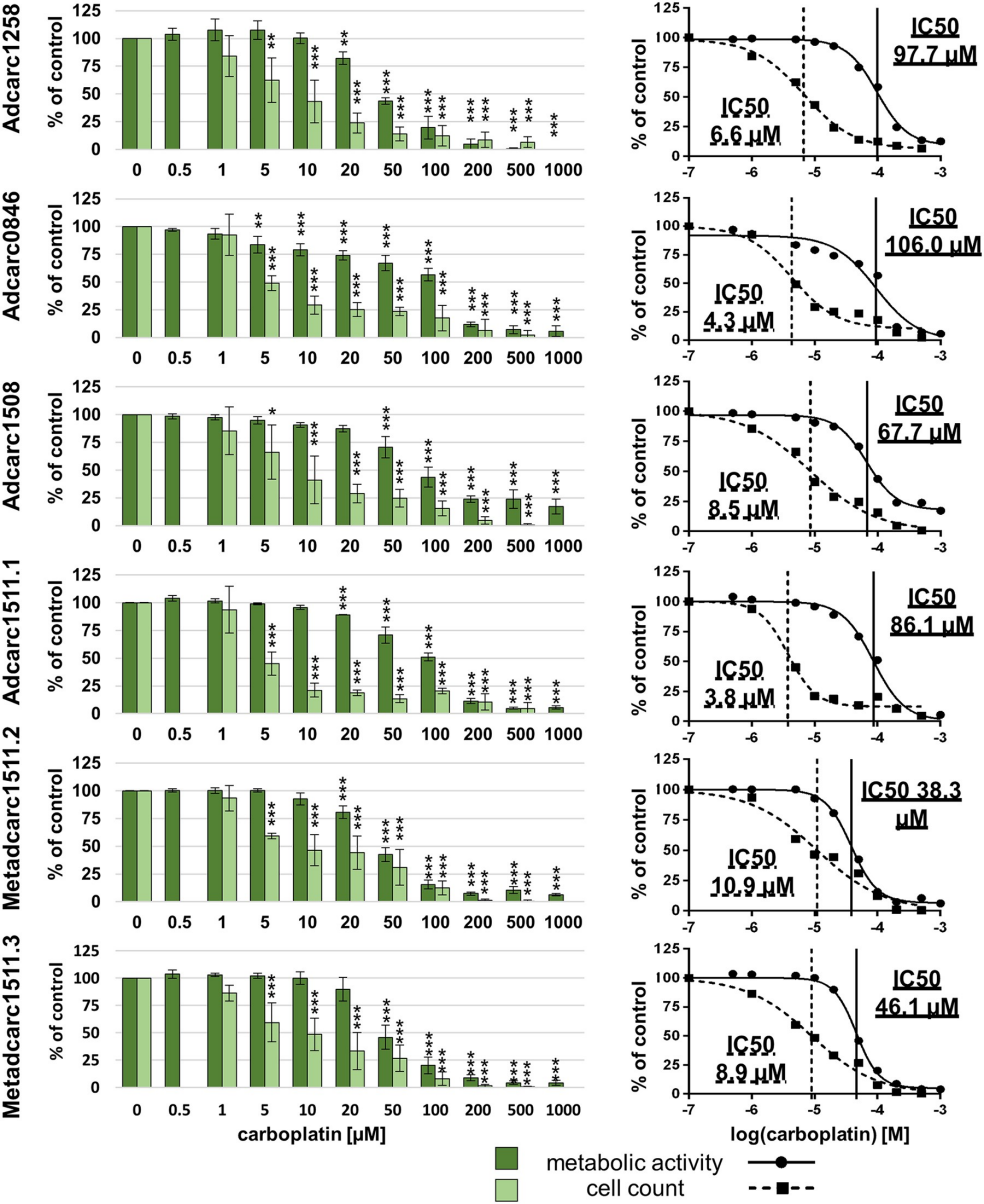
Metabolic activities and cell counts of PAC cell lines after 72 h incubation with carboplatin. Means and standard deviation are displayed, n = 3 for metabolic activity, n = 4 for cell count, significances are referred to controls. *p < 0.05, p** < 0.01 and p*** <0.01.

**Fig 6:**
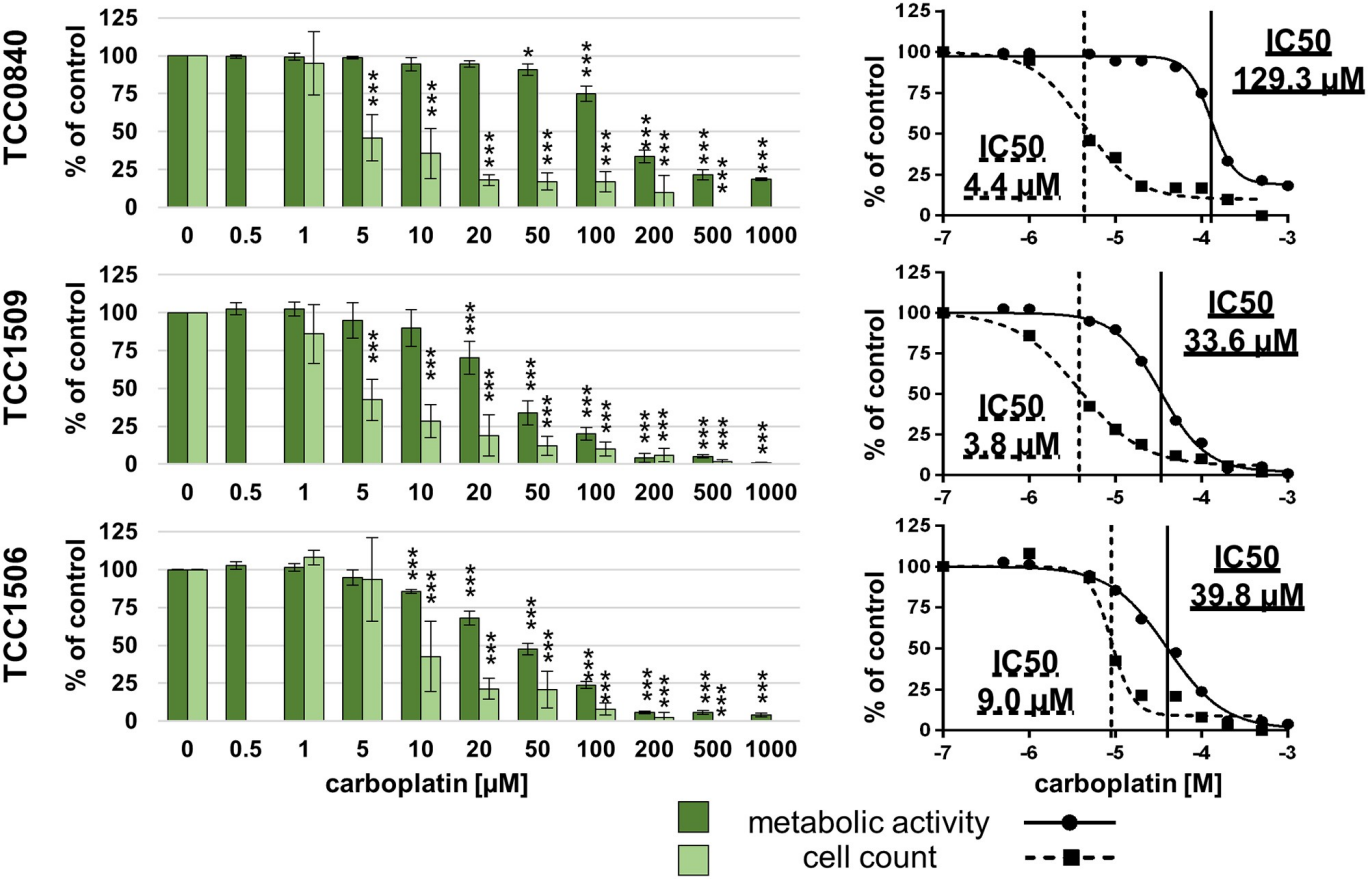
Metabolic activities and cell counts of TCC cell lines after 72 h incubation with carboplatin. Means and standard deviation are displayed, n = 3 for metabolic activity, n = 4 for cell count, significances are referred to controls. *p < 0.05, p** < 0.01 and p*** <0.01.

Carboplatin metabolic activity IC50 values varied between 33.6 and 129.3 μM in all cell lines with Metadcarc1511.2 and Metadcarc1511.3 ranging in the lower half (Table 4). Reductions of metabolic activities below 18% could be achieved in all cell lines at 1000 μM carboplatin (Figs 5 and 6).

Cell count IC50 values for carboplatin ranged from 3.8 μM in TCC1509 and Adcarc1511.1 to 10.9 μM in Metadcarc1511.2 (Table 4). Reductions of total cell numbers below seven percent could be achieved in all cell lines at 500 μM carboplatin.

### Induction of apoptosis by doxorubicin and carboplatin

**Fig 7:**
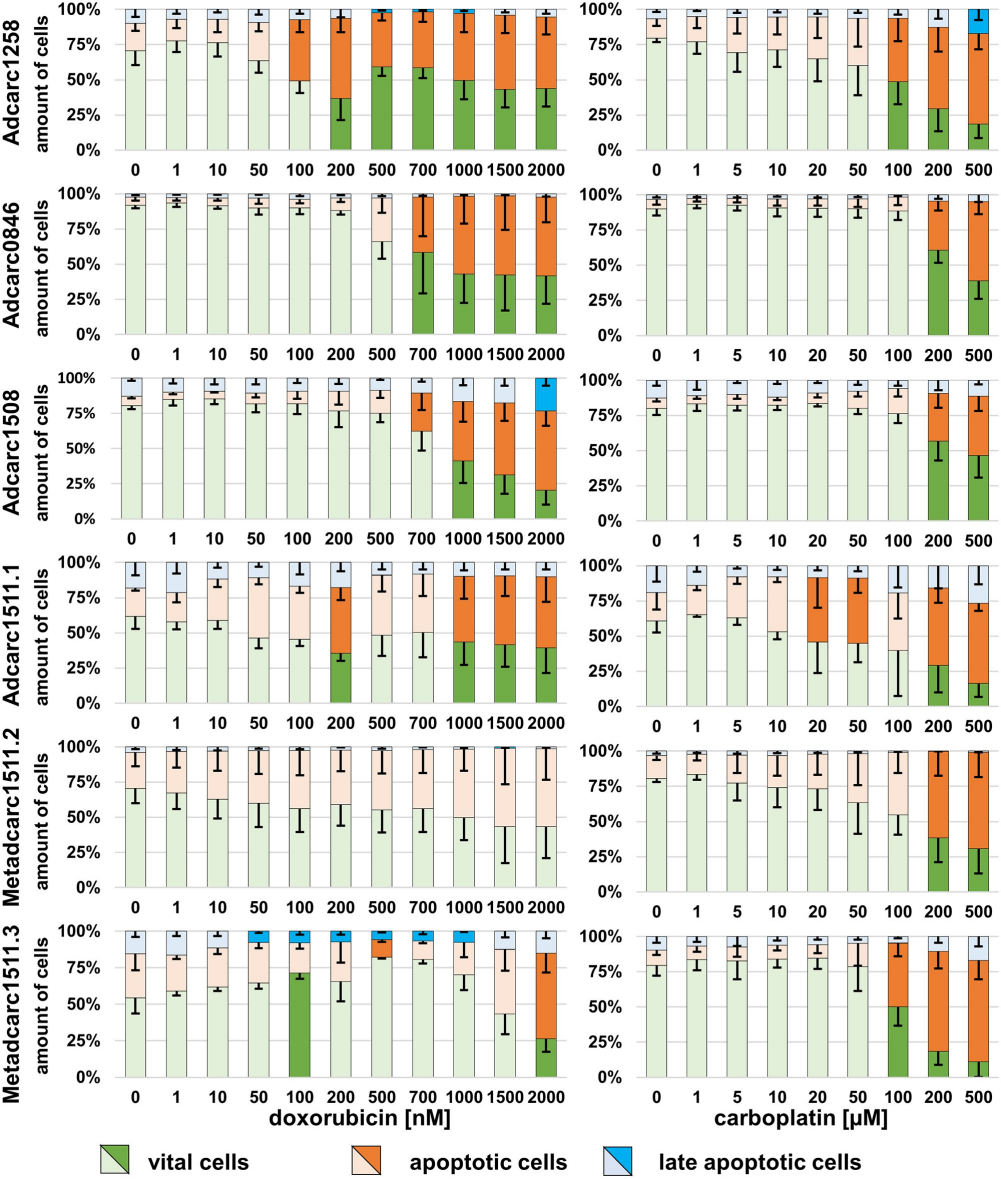
Induction of apoptosis in PAC cell lines after 72 h incubation with doxorubicin or carboplatin. Vital (green), apoptotic (orange) and late apoptotic (blue) proportions of PAC cell lines; means and standard deviations are displayed. Dark colours display significant differences referred to untreated controls. *p < 0.05; n = 4.

**Fig 8:**
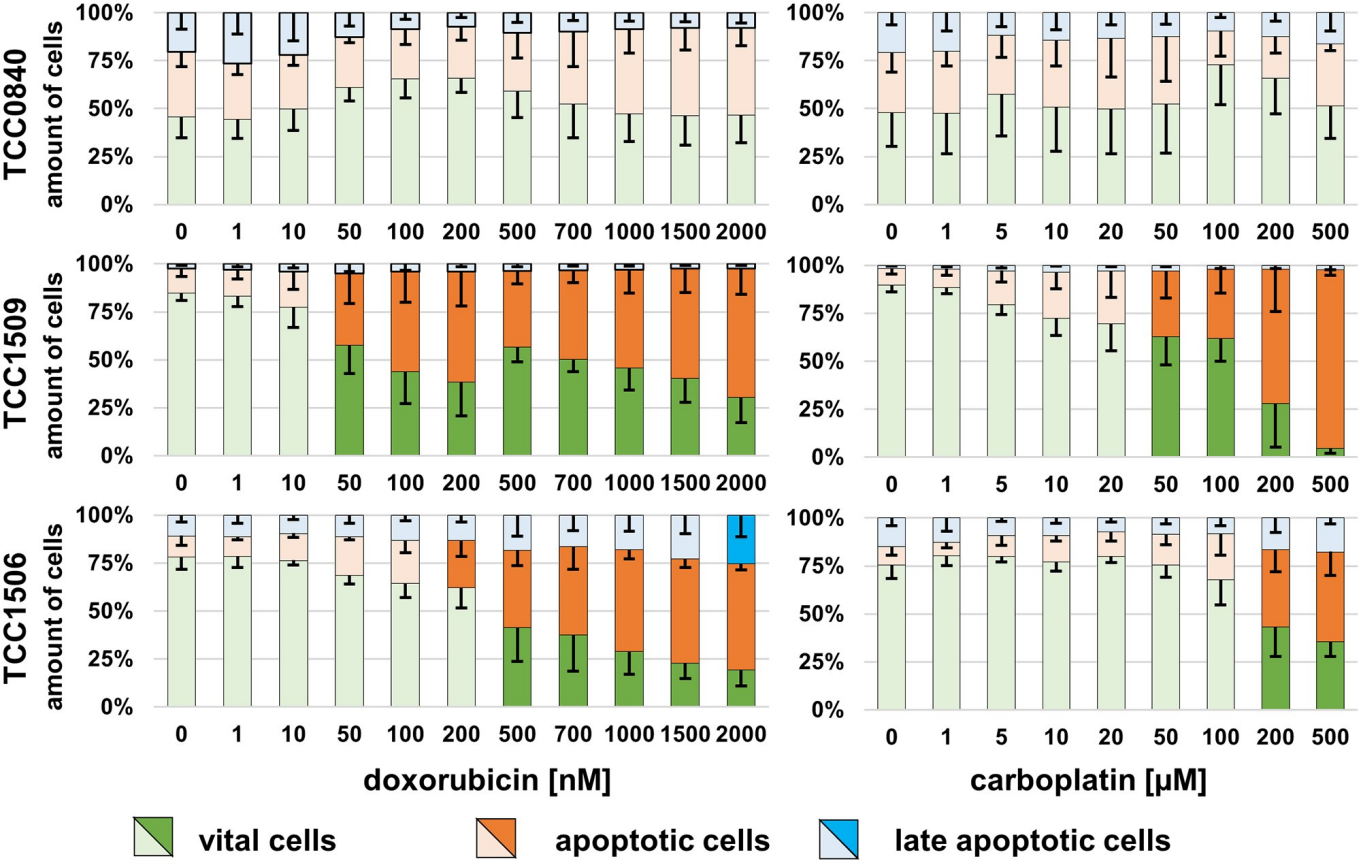
Induction of apoptosis in TCC cell lines after 72 h incubation with doxorubicin or carboplatin. Vital (green), apoptotic (orange) and late apoptotic (blue) proportions of TCC cell lines; means and standard deviations are displayed. Dark colours display significant differences referred to untreated controls. *p < 0.05; n = 4.

Doxorubicin and carboplatin induced dose-dependent increases of apoptotic cell proportions and decreases of vital cells in nearly all cell lines (Figs 7 and 8). No significant changes were observed in TCC0840 in general and Metadcarc1511.2 treated with doxorubicin (Figs 7 and 8). In TCC0840, proportions of vital cells were below 50% in controls and high amounts of apoptotic cells were observed in all samples (Fig 8). Exposed to doxorubicin, TCC1509 was the first cell line reacting with increased proportions of apoptotic cells, followed by Adcarc1258 at 100 nM. Metadcarc1511.3 changed proportions of vital and apoptotic cells at 2000 nM doxorubicin.

After 72 h exposure to carboplatin, Adcarc1511.1 was the cell line reacting first with increased apoptosis rates at 20 μM. At 200 and 500 μM carboplatin, all cell lines except from TCC0840 showed increased apoptotic rates.

### Inhibition of COX-2

Metabolic activities and total cell numbers did not decrease in any cell line treated with meloxicam in both concentrations (S2 Fig).

**Fig 9:**
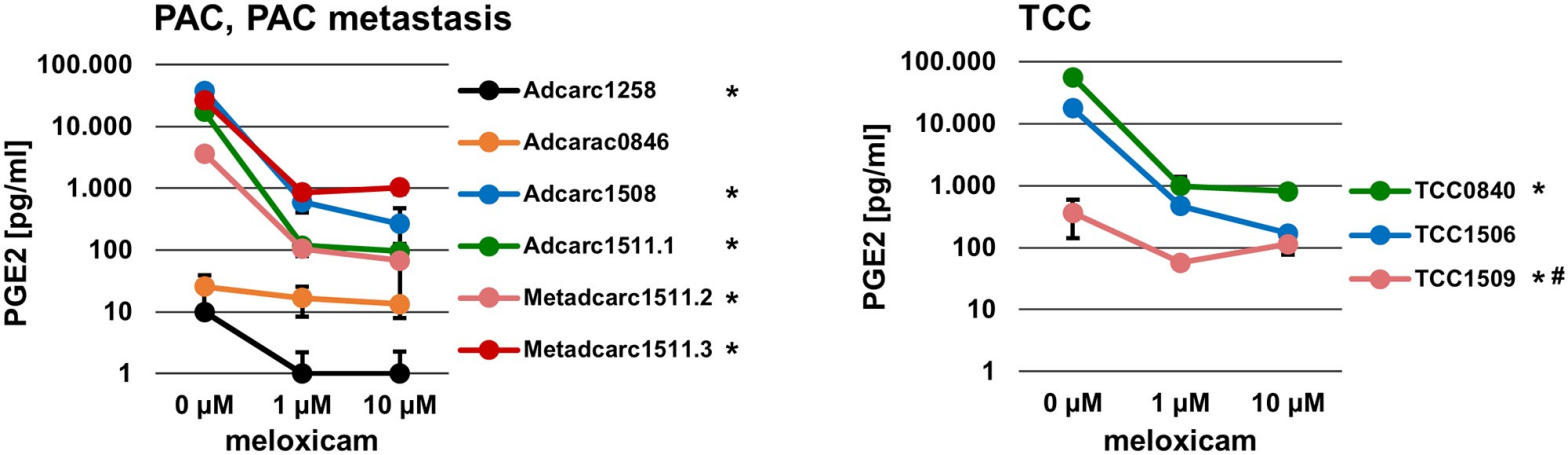
PGE2 production of cell lines after 24 h incubation with meloxicam. Measured in duplicates by competitive ELISA; * PGE2 production in pg/ml medium in both meloxicam concentrations, compared to 0 μM DMSO control (Dunnett’s test for multiple comparisons), # additional significant difference between 1 and 10 μM in cell line TCC1509 (Student’s t-test), p < 0.05.

Basal PGE2 concentrations in medium ranged beyond 1000 pg/ml in all cell lines except from Adcarc1258, Adcarc0846 and TCC1509 (Fig 9). Meloxicam decreased PGE2 production in both concentrations in all cell lines except from Adcarc0846 and TCC1509. TCC1506 was the only cell line, in which the reduction achieved with 1 μM meloxicam could be enhanced by a tenfold higher concentration.

## Discussion

The presented study provides nine thoroughly characterized cell lines of canine lower urinary tract cancer. Therefore, combined in a panel, these cell lines offer a great opportunity to evaluate therapeutic strategies in any case of lower urinary tract cancer in dogs, including TCC located in the prostate and PAC metastasis.

When comparing initial characterizations of different human [1,45,67,68] and canine [16,33,34,36–39,46] PAC and TCC cell lines, a number of substantial features were commonly profiled. Cell morphology, doubling time and growth behavior are important and useful information researchers need for experimental design [46,69]. Medical patient data and resistance analysis to classical chemotherapeutic drugs allow assessing the malignancy, multiresistances and the metastatic potential [38,68,70], though chemoresistances are often analyzed in further studies [70–72]. However, only three K9TCC cell lines [46] and eight of the herein profiled cell lines were immunohistochemically characterized in direct comparison to the respective original tumor tissue. For all those cell lines, the majority of immunohistochemical marker expressions were preserved during culturing process, suggesting that these cell lines generally are suitable models of the respective tumors they were derived from. However, all herein characterized cell lines also displayed some immunohistochemical differences to the respective tumor tissue indicating that they have undergone individual changes induced by subculturing.

Some of the previously mentioned human and canine PAC and TCC cell lines were characterized in early and some in later passages. However, subculturing-induced gene expression changes were reported to happen within the first ten passages [73], particularly losses of claudin gene expressions [5]. As stable *in vitro* models are required for repeatable results in further experiments, cell lines were herein characterized in later passages.

Metastasis research is an important part in human and veterinary cancer research. Various studies were carried out using metastasis-derived prostate cancer cell lines like LNCaP and PC-3 [74] or metastasis inducing cell lines like Leo, Probasco, Ace-1, DCP-1, PC-3 or LNCaP [75,76] in mouse xenograft or dog allograft models. To our knowledge, there are no reported paired cell lines derived from primary tumor and metastasis of spontaneously occurring prostate cancer from the same patient. As confirmed by immunohistochemistry, several cellular features remained conserved in metastases Ln4.2 and Ln4.3 compared to the respective primary tumor and in derived cell lines compared to the respective tissues. However, differences between Adcarc1511.1 and the paired metastasis-derived cell lines were demonstrated in increased cell size and prolonged doubling times. Contrary to carboplatin response, both metastasis cell lines showed additional chemoresistance towards doxorubicin compared to Adcarc1511.1. Disseminated tumor cells have to overcome numerous adverse conditions when establishing distant metastasis and were consistently described to be more resistant to microenvironmental apoptosis-inducing conditions and chemotherapeutic treatments [31,32].

The broad range of the herein analyzed cell lines’ IC50 values for doxorubicin are comparable with human prostate and bladder cancer cells [77] and with canine mammary carcinoma cells [78]. Concerning carboplatin, the herein characterized TCC cell lines are more sensitive than other canine TCC cell lines [70] and more comparable to human prostate cancer cells [71]. Adcarc1258 and TCC0840 resemble models for multidrug resistance, as they were rather resistant to both doxorubicin and carboplatin.

In most cell lines, cell counts decreased before apoptotic proportions of cells increased, indicating that doxorubicin and carboplatin first inhibit proliferation and induce apoptosis at higher concentrations. However, in Adcarc1258 after exposure to doxorubicin and in TCC1509 after exposure to both drugs, apoptosis began at lower concentrations compared to the other cell lines, which makes these cell lines more sensitive to apoptosis inducing stimuli.

Exposed to high concentrations of doxorubicin, still a number of cells survived in both metastasis-derived cell lines and in TCC0840. Especially in those cell lines, apoptotic proportions of cells did not increase until 2000 nM, indicating that doxorubicin inhibits proliferation, but does not induce apoptosis. Apoptotic proportions of TCC0840 were relatively high in controls and in all treated samples. It remains uncertain if the detachment of cells or the staining procedure induced apoptosis in this cell line or if a certain proportion of this cell line constantly underwent apoptosis. Changes of vital and apoptotic proportions of cells in cell lines Adcarc1511.1 and Metadcarc1511.3 that were observed at 200 and 100 μM doxorubicin but not at higher concentrations might be of artificial origin. Prominently in both metastasis-derived cell lines after treatment with doxorubicin and in Adcarc1258, TCC0840, Adcarc0846 and Adcarc1511.1 after carboplatin exposure, total cell numbers decreased much faster than metabolic activities. Various transporters and other avoidance mechanisms are involved in doxorubicin and carboplatin resistance [79,80]. Assumingly, cells that did not instantly undergo apoptosis when exposed to low doses of doxorubicin or carboplatin upregulated resistance mechanisms resulting in a higher metabolism of the single cell. Consequently, the higher metabolism of struggling cells masked the effect of doxorubicin and carboplatin and lead to a delayed decrease of metabolic activity compared to cell numbers.

Daily administered COX-2 inhibitors increase survival times in dogs with prostate and bladder tumors [8,10,81]. However, the steady state plasma concentration of 1 μM meloxicam in dogs [66] had no impact on proliferation and metabolic activity in all analyzed cell lines *in vitro*, which was also demonstrated for human prostate cancer cell lines [82] and canine osteosarcoma and mammary cancer cell lines [83–85]. Consistent with other canine TCC cell lines, basal PGE2 production was relatively high, compared to cell lines of other tumor entities [86], supporting therapeutic efficiency of COX-2 inhibitors. Further studies are needed to investigate effects of COX-2 inhibitors on features like migration and invasion, as they were shown for canine mammary cancer [84,85]. In Adcarc0846 and TCC1509, in which COX-2 protein level was the lowest, PGE2 production was not influenced by meloxicam. Mutations or other modifications might be reasons for the steady active enzyme in those cases. The decrease of PGE2 production in the remaining seven cell lines was enhanced by 10 μM compared to 1 μM meloxicam in only one cell line. Drastically increased drug concentrations that inhibited metabolic activity in other *in vitro* studies [83,87] might have additional effects other than those inhibiting COX-2 enzyme activity.

Due to a short half-life of its wild type, p53 is almost exclusively detectable in accumulated and mutated forms by immunohistochemistry [13,14]. Mutations of important tumor suppressor p53 are correlated with progression, poor differentiation and metastasis in human PAC and TCC [18,88,89]. Dhawan et al. reported an overexpression of p53 in three out of eight canine TCC cell lines [16], which is consistent with this study detecting p53 immunoreactivity in the original tissue B7 from one patient and corresponding cell line TCC1506 out of three canine TCC cell lines. In the particular case of B7, the negative prognostic value of p53 could be confirmed, as the dog died from a metastatic and chemoresistant tumor recurrence one year after surgery. Despite the negative result for all highly malignant PAC cases, *p53* mutation cannot be excluded. Specific mutations affecting the binding site of the herein used antibody might prevent it from binding to p53 [14].

Epithelial to mesenchymal transition is a common pathological process in canine metastatic prostate cancer [90,91]. It is suggested that the analyzed cell lines and respective tumor tissues show features of epithelial to mesenchymal transition, as they express both epithelial (pan-CK, CK7, CK8/18, E-Cad) and mesenchymal (vimentin, Calp) markers concurrently [6,90]. Consequently, epithelial to mesenchymal transition is likely to be advanced in Adcarc1258, as its primary tumor does only express E-Cad as epithelial marker and even this feature gets lost in the cell line. Adaption processes to culturing conditions or clonal selection of small proportions of tumor cells [6,92,93] may explain the further mesenchymal transition of cell lines Adcarc1508, TCC1509, Adcarc1511.1, Metadcarc1511.2 and Metadcarc1511.3, which is characterized by gains of vimentin and losses of pan-CK expression.

All tumors apart from P1 possess basal cell features by expressing pan-CK and luminal cell characteristics by staining positive for CK7 and CK8/18, which supports the involvement of basal and luminal cells in canine prostate cancerogenesis [94,95]. UPIII protein, usually expressed by urothelial cells [94], was not detected in any of the samples, which is contrary to other studies with large numbers of canine non-neoplastic prostate and neoplastic bladder samples [59,94]. This may be caused by areal UPIII expression losses in high grade TCCs [96]. None of the analyzed immunohistochemical markers is appropriate to distinguish between PAC and TCC in this study.

Like in the human prostate cancer cell line LNCaP [68], Adcarc1258 and TCC1509 had low anchoring potential and did not establish uniform monolayers in culture. Instead, cells tended to form aggregates with increasing cell density, which detached after further cultivation. As previously shown by our group, Adcarc1258 forms spheroids in suspension culture that are more resistant towards doxorubicin [55]. This behavior, which might also be present in TCC1509, can be useful for investigation of resistance mechanisms or stem cell features. In addition to the characteristics assessed within this study, further features are already available for some of the cell lines. For Adcarc1258 and TCC0840, karyotype analyses have been published [35,51] and the expression of claudin genes has been assessed for Adcarc1258, TCC0840 and Adcarc0846 [57]. Adcarc1258 induces tumors when injected in immunodeficient mice [52]. Additional information about doxorubicin resistance, stem cell marker gene expressions and a GFP-transfected subline of Adcarc1258 are accessible [54,55].

Transcriptomic analysis of canine PAC tissues referred to non-cancerous controls will be the next step to identify upregulated cancer-specific targets for promising inhibitors [80]. Further gene expression analysis of cell lines will reveal which cell line conserves these targets and therefore appears to be the best model. Studies applying these specific inhibitors or combined therapeutics in suitable cell lines will follow to select effective treatments for canine lower urinary tract cancer.

## Conclusions

Summarized, this thorough characterization enriches canine lower urinary tract cancer research with nine valuable *in vitro* tools that are immunohistochemically close to the respective tumor tissue. Adcarc1511.1, Metadcarc1511.2 and Metadcarc1511.3 provide the unique opportunity to investigate functional cellular features associated with canine PAC metastasis, as they were derived from primary tumor and metastasis of the same patient. TCC0840 and TCC1509 represent the uncertain percentage of TCC with invasion or emergence in prostate tissue. Adcarc1258 and TCC0840 represent multi-resistant canine PAC and TCC, while TCC1506 and TCC1509 are chemosensitive. Both metastasis derived cell lines Metadcarc1511.2 and Metadcarc1511.3 are resistant to apoptotic effects induced by doxorubicin, but susceptive to carboplatin treatment.

## Supporting information

S1 Table Medical patient data, tissue samples and cell lines. m = male; f = female; n = neutered; original tissue (P = Prostate, B = urinary bladder, Ln = lymph node); cell line names are explained as institution (Tiho = University of Veterinary Medicine Hannover); species (D = dog); tissue origin (Pro = prostate; Urt = urinary tract (urinary bladder)); diagnosis (Adcarc = adenocarcinoma; Carc = carcinoma; Metadcarc = metastasis of an adenocarcinoma); abbreviations of cell lines written in bold; none = cell lines have not been published yet; n.a. = tissue of patient no. 5 is missing, as the patient owners declined surgery and necropsy; *diagnosis by cytology of cells obtained by fine needle aspiration biopsy; **in total remission after a combination protocol of vincristine, asparaginase, cyclophosphamide, doxorubicin, prednisone and lomustin.
(DOCX)

S2 Table Antibodies used for immunohistochemistry. (DOCX)

S1 Fig Exclusion of fibroblasts by collagen VI immunofluorescence (red), nuclei are counterstained with DAPI (blue). (TIFF)

S2 Fig Metabolic activities and cell counts after 72 h incubation with meloxicam, n = 3. (TIF)

S1 Video Growth behavior of Adcarc1258 over 72 h. (MP4)

S2 Video Growth behavior of Adcarc0846 over 72 h. (MP4)

S3 Video Growth behavior of Adcarc1508 over 72 h. (MP4)

S4 Video Growth behavior of Adcarc1511.1 over 72 h. (MP4)

S5 Video Growth behavior of Metadcarc1511.2 over 72 h. (MP4)

S6 Video Growth behavior of Metadcarc1511.3 over 72 h. (MP4)

S7 Video Growth behavior of TCC0840 over 72 h. (MP4)

S8 Video Growth behavior of TCC1509 over 72 h. (MP4)

S9 Video Growth behavior of TCC1506 over 72 h. (MP4)

## References

[1] Cunningham D, You Z. In vitro and in vivo model systems used in prostate cancer research. J Biol Methods. 2015; 2:1–28. 10.14440/jbm.2015.63 PMC4487886 26146646

[2] Krook L. a Statistical Investigation of Carcinoma in the Dog. Acta Pathol. Microbiol. Scand. 1954; 407–22. 10.1111/j.1699-0463.1954.tb00886.x 13206777

[3] Bell FW, Klausner JS, Hayden DW, Feeney DA, Johnston SD. Clinical and pathologic features of prostatic adenocarcinoma in sexually intact and castrated dogs: 31 cases (1970–1987). J. Am. Vet. Med. Assoc. 1991; 1623–30. 1778750

[4] Ertel A, Verghese A, Byers SW, Ochs M, Tozeren A. Pathway-specific differences between tumor cell lines and normal and tumor tissue cells. Mol Cancer. 2006; 5:55 10.1186/1476-4598-5-55 17081305 PMC1635729

[5] Hammer SC, Becker A, Rateitschak K, Mohr A, Ripoli FL, Hennecke S, et al Longitudinal claudin gene expression analyses in canine mammary tissues and thereof derived primary cultures and cell lines. Int J Mol Sci. 2016; 17 10.3390/ijms17101655 27690019 PMC5085688

[6] Granados-Soler JL, Junginger J, Hewicker-Trautwein M, Bornemann-Kolatzki K, Beck J, Brenig B, et al TiHo-0906: a new feline mammary cancer cell line with molecular, morphological, and immunocytological characteristics of epithelial to mesenchymal transition. Sci Rep. 2018; 8:1–17. 30185896 10.1038/s41598-018-31682-1 PMC6125410

[7] LeRoy BE, Northrup N. Prostate cancer in dogs: Comparative and clinical aspects. Vet J. 2009; 180:149–62. 10.1016/j.tvjl.2008.07.012 18786842

[8] Fulkerson CM, Knapp DW. Management of transitional cell carcinoma of the urinary bladder in dogs: A review. Vet J. 2015; 205:217–25. 10.1016/j.tvjl.2015.01.017 25747698

[9] Cannon CM, Allstadt SD. Lower Urinary Tract Cancer. Vet Clin North Am—Small Anim Pract. 2015; 45:807–24. 10.1016/j.cvsm.2015.02.008 25824392

[10] Sorenmo KU, Goldschmidt MH, Shofer FS, Goldkamp C, Ferracone J. Evaluation of cyclooxygenase-1 and cyclooxygenase-2 expression and the effect of cyclooxygenase inhibitors in canine prostatic carcinoma. Vet Comp Oncol. 2004; 2:13–23. 10.1111/j.1476-5810.2004.00035.x 19379307

[11] Ravicini S, Baines SJ, Taylor A, Amores-Fuster I, Mason SL, Treggiari E. Outcome and prognostic factors in medically treated canine prostatic carcinomas: A multi-institutional study. Vet Comp Oncol. 2018; 16:450–8. 10.1111/vco.12400 29806232

[12] Rivera-Calderón LG, Fonseca-Alves CE, Kobayashi PE, Carvalho M, Drigo SA, de Oliveira Vasconcelos R, et al Alterations in PTEN, MDM2, TP53 and AR protein and gene expression are associated with canine prostate carcinogenesis. Res Vet Sci. 2016; 106:56–61. 10.1016/j.rvsc.2016.03.008 27234536

[13] Esrig D, Spruck CH, Nichols PW, Chaiwun B, Steven K, Groshen S, et al p53 Nuclear protein accumulation correlates with mutations in the p53 gene, tumor grade, and stage in bladder cancer. Am J Pathol. 1993; 143:1389–97. 7901994 PMC1887166

[14] Zacchetti A, van Garderen E, Rutteman GR. Immunohistochemical evaluation of p53 expression with different antibodies in malignant canine tumours with or without p53 gene mutation. Vet Comp Oncol. 2007; 5:108–18. 10.1111/j.1476-5829.2006.00120.x 19754794

[15] Kirpensteijn J, Kik M, Teske E, Rutteman GR. TP53 gene mutations in canine osteosarcoma. Vet Surg. 2008; 37:454–60. 10.1111/j.1532-950X.2008.00407.x 18986312

[16] Dhawan D, Ramos-Vara JA, Stewart JC, Zheng R, Knapp DW. Canine invasive transitional cell carcinoma cell lines: In vitro tools to complement a relevant animal model of invasive urinary bladder cancer. Urol Oncol Semin Orig Investig. 2009; 27:284–92. 10.1016/j.urolonc.2008.02.015 18562222

[17] Suárez-Bonnet A, Herráez P, Aguirre M, Suárez-Bonnet E, Andrada M, Rodríguez F, et al Expression of cell cycle regulators, 14-3-3σ and p53 proteins, and vimentin in canine transitional cell carcinoma of the urinary bladder. Urol Oncol Semin Orig Investig. 2015; 33:332.e1–332.e7. 10.1016/j.urolonc.2015.04.006 25979650

[18] Pagliarone S, Frattone L, Pirocchi V, Della Salda L , Palmieri C. Altered expression of p53, but not Rb, is involved in canine prostatic carcinogenesis. Res Vet Sci. 2016; 105:195–9. 27033932 10.1016/j.rvsc.2016.02.022

[19] Palmieri C, Grieco V. Proposal of Gleason-like grading system of canine prostate carcinoma in veterinary pathology practice. Res Vet Sci. 2015; 103:11–5. 10.1016/j.rvsc.2015.09.004 26679789

[20] Palmieri C, Foster R, Grieco V, Fonseca-Alves CE, Wood G, Culp WT, et al Pathology of the canine prostate: Recommendations of an oncology/pathology working group. J Comp Pathol. 2019; 166:100 10.1016/j.jcpa.2018.10.003

[21] Lai CL, Van Den Ham R, Van Leenders G, Van Der Lugt J, Mol JA, Teske E. Histopathological and immunohistochemical characterization of canine prostate cancer. Prostate. 2008; 68:477–88. 10.1002/pros.20720 18196537

[22] Knapp DW, Henry CJ, Widmer WR, Tan KM, Moore GE, Ramos-Vara JA, et al Randomized Trial of Cisplatin versus Firocoxib versus Cisplatin/Firocoxib in Dogs with Transitional Cell Carcinoma of the Urinary Bladder. J Vet Intern Med. 2013; 27:126–33. 10.1111/jvim.12013 23205923

[23] Robat C, Burton J, Thamm D, Vail D. Retrospective evaluation of doxorubicin-piroxicam combination for the treatment of transitional cell carcinoma in dogs. J Small Anim Pract. 2013; 54:67–74. 10.1111/jsap.12009 23286739

[24] Sun F, Báez-Díaz C, Sánchez-Margallo FM. Canine prostate models in preclinical studies of minimally invasive interventions: Part I, canine prostate anatomy and prostate cancer models. Transl Androl Urol. 2017; 6:538–46. 10.21037/tau.2017.03.61 28725597 PMC5503961

[25] Fonseca-Alves CE, Kobayashi PE, Palmieri C, Laufer-Amorim R. Investigation of c-KIT and Ki67 expression in normal, preneoplastic and neoplastic canine prostate. BMC Vet Res. 2017; 13:380 10.1186/s12917-017-1304-0 29207991 PMC5718037

[26] Palmieri C, Foster RA, Grieco V, Fonseca-Alves CE, Wood GA, Culp WTN, et al Histopathological Terminology Standards for the Reporting of Prostatic Epithelial Lesions in Dogs. J Comp Pathol. 2019; 171:30–7. 10.1016/j.jcpa.2019.07.005 31540623

[27] LeRoy BE, Nadella MVP, Toribio RE, Leav I, Rosol TJ. Canine prostate carcinomas express markers of urothelial and prostatic differentiation. Vet Pathol. 2004; 41:131–40. 10.1354/vp.41-2-131 15017026

[28] Heilmann RM, Wright ZM, Lanerie DJ, Suchodolski JS, Steiner JM. Measurement of urinary canine S100A8/A9 and S100A12 concentrations as candidate biomarkers of lower urinary tract neoplasia in dogs. J Vet Diagnostic Investig. 2014; 26:104–12. 10.1177/1040638713516625 24398905

[29] Heilmann RM, McNiel EA, Grützner N, Lanerie DJ, Suchodolski JS, Steiner JM. Diagnostic performance of the urinary canine calgranulins in dogs with lower urinary or urogenital tract carcinoma. BMC Vet Res. 2017; 13:1–11. 28431528 10.1186/s12917-017-1032-5 PMC5401473

[30] Powe JR, Canfield PJ, Martin PA. Evaluation of the cytologic diagnosis of canine prostatic disorders. Vet Clin Pathol. 2004; 33:150–4. 10.1111/j.1939-165x.2004.tb00365.x 15334350

[31] Eccles SA, Welch DR. Metastasis: recent discoveries and novel treatment strategies. Lancet (London, England). 2007; 369:1742–57. 10.1016/S0140-6736(07)60781-8 17512859 PMC2214903

[32] Wade CA, Kyprianou N. Profiling Prostate Cancer Therapeutic Resistance. Int J Mol Sci. 2018; 19 10.3390/ijms19030904 29562686 PMC5877765

[33] Eaton CL, Pierrepoint CG. Growth of a spontaneous canine prostatic adenocarcinoma in vivo and in vitro: isolation and characterization of a neoplastic prostatic epithelial cell line, CPA 1. Prostate. 1988; 12:129–43. 10.1002/pros.2990120204 3368402

[34] Martn JJ, Martn R, Codesal J, Fraile B, Paniagua R, Santamara L. In vivo model mimicking natural history of dog prostate cancer using DPC-1, a new canine prostate carcinoma cell line. Prostate. 2001; 46:2–10. 10.1002/1097-0045(200101)46:1<2::aid-pros1002>3.0.co;2-5 11170126

[35] Winkler S, Murua Escobar H, Eberle N, Reimann-Berg N, Nolte I, Bullerdiek J. Establishment of a cell line derived from a canine prostate carcinoma with a highly rearranged karyotype. J Hered. 2005; 96:782–5. 10.1093/jhered/esi085 15994418

[36] LeRoy BE, Thudi NK, Nadella MVP, Toribio RE, Tannehill-Gregg SH, van Bokhoven A, et al New Bone Formation and Osteolysis by a Metastatic, Highly Invasive Canine Prostate Carcinoma Xenograft. Prostate. 2006;: 1213–22. 10.1002/pros.20408 16683269

[37] Thudi NK, Shu ST, Martin CK, Lanigan LG, Nadella MVP, Van Bokhoven A, et al Development of a brain metastatic canine prostate cancer cell line. Prostate. 2011; 71:1251–63. 10.1002/pros.21341 21321976 PMC3139788

[38] Simmons JK, Dirksen WP, Hildreth BE, Dorr C, Williams C, Thomas R, et al Canine prostate cancer cell line (Probasco) produces osteoblastic metastases in vivo. Prostate. 2014; 74:1251–65. 10.1002/pros.22838 25043424 PMC4216720

[39] Azakami D, Nakahira R, Kato Y, Michishita M, Kobayashi M, Onozawa E, et al The canine prostate cancer cell line CHP-1 shows over-expression of the co-chaperone small glutamine-rich tetratricopeptide repeat-containing protein α. Vet Comp Oncol. 2017; 15:557–62. 10.1111/vco.12199 26762899

[40] Cornell KK, Bostwick DG, Cooley DM, Hall G, Harvey HJ, Hendrick MJ, et al Clinical and pathologic aspects of spontaneous canine prostate carcinoma: A retrospective analysis of 76 cases. Prostate. 2000; 45:173–83. 10.1002/1097-0045(20001001)45:2<173::aid-pros12>3.0.co;2-r 11027417

[41] Hazzah TN, Kass PH, Brodsky EM, Elpiner AK, Silver ML, Buote NJ, et al Evaluation of mitoxantrone with piroxicam as first line therapy for carcinomas of the prostate in dogs. Int J Appl Res Vet Med. 2013; 11:16–24.

[42] Cekanova M, Rathore K. Animal models and therapeutic molecular targets of cancer: utility and limitations. Drug Des Devel Ther. 2014; 8:1911–21. 10.2147/DDDT.S49584 25342884 PMC4206199

[43] Shapiro SG, Knapp DW, Breen M. A cultured approach to canine urothelial carcinoma: molecular characterization of five cell lines. Canine Genet Epidemiol. 2015; 2:15 10.1186/s40575-015-0028-3 26401343 PMC4579363

[44] Ramsey SA, Xu T, Goodall C, Rhodes AC, Kashyap A, He J, et al Cross-species analysis of the canine and human bladder cancer transcriptome and exome. Genes Chromosom Cancer. 2017; 56:328–43. 10.1002/gcc.22441 28052524

[45] Ringuette-Goulet C, Bolduc S, Pouliot F. Modeling human bladder cancer. World J Urol. 2018; 36:1–8. 10.1007/s00345-018-2369-5 29948049

[46] Rathore K, Cekanova M. Animal model of naturally occurring bladder cancer: Characterization of four new canine transitional cell carcinoma cell lines. BMC Cancer. 2014; 14:465 10.1186/1471-2407-14-465 24964787 PMC4082678

[47] Shapiro SG, Raghunath S, Williams C, Motsinger-Reif AA, Cullen JM, Liu T, et al Canine urothelial carcinoma: genomically aberrant and comparatively relevant. Chromosom Res. 2015; 23:311–31. 10.1007/s10577-015-9471-y 25783786 PMC4501039

[48] Valli VE, Norris A, Jacobs RM, Laing E, Withrow S, Macy D, et al Pathology of canine bladder and urethral cancer and correlation with tumour progression and survival. J Comp Pathol. 1995; 113:113–30. 10.1016/s0021-9975(05)80027-1 8543669

[49] Knapp DW, Glickman NW, Denicola DB, Bonney PL, Lin TL, Glickman LT. Naturally-occurring canine transitional cell carcinoma of the urinary bladder: A relevant model of human invasive bladder cancer. Urol Oncol. 2000; 5:47–59. 10.1016/s1078-1439(99)00006-x 21227289

[50] Palmieri C, Lean FZ, Akter SH, Romussi S, Grieco V. A retrospective analysis of 111 canine prostatic samples: Histopathological findings and classification. Res Vet Sci. 2014; 97:568–73. 10.1016/j.rvsc.2014.11.006 25468798

[51] Reimann-Berg N, Willenbrock S, Murua Escobar H, Eberle N, Gerhauser I, Mischke R, et al Two new cases of polysomy 13 in canine prostate cancer. Cytogenet Genome Res. 2011; 132:16–21. 10.1159/000317077 20668368

[52] Fork MA, Murua Escobar H, Soller JT, Sterenczak KA, Willenbrock S, Winkler S, et al Establishing an in vivo model of canine prostate carcinoma using the new cell line CT1258. BMC Cancer. 2008; 8:240 10.1186/1471-2407-8-240 18706092 PMC2527616

[53] Moulay M, Liu WEN, Willenbrock S, Sterenczak KA, Carlson R, Ngezahayo A, et al Evaluation of Stem Cell Marker Gene Expression in Canine Prostate Carcinoma- and Prostate Cyst-derived Cell Lines. Anticancer Res. 2013; 33:5421–31. 24324078

[54] Willenbrock S, Wagner S, Reimann-Berg N, Moulay M, Hewicker-Trautwein M, Nolte I, et al Generation and characterisation of a canine EGFP-HMGA2 prostate cancer in vitro model. PLoS One. 2014; 9:1–12. 10.1371/journal.pone.0098788 24914948 PMC4051699

[55] Liu W, Moulay M, Willenbrock S, Roolf C, Junghanss C, Ngenazahayo A, et al Comparative characterization of stem cell marker expression, metabolic activity and resistance to doxorubicin in adherent and spheroid cells derived from the canine prostate adenocarcinoma cell line CT1258. Anticancer Res. 2015; 35:1917–27. 25862843

[56] Wagner S, Maibaum D, Pich A, Nolte I, Escobar HM. Verification of a canine PSMA (FolH1) antibody. Anticancer Res. 2015; 35:145–8. 35/1/145 [pii] 25550545

[57] Hammer SC, Nagel S, Junginger J, Hewicker-Trautwein M, Wagner S, Heisterkamp A, et al Claudin-1, -3, -4 and -7 gene expression analyses in canine prostate carcinoma and mammary tissue derived cell lines. Neoplasma. 2016; 63:231–8. 10.4149/208_150924N505 26774145

[58] Harting T, Stubbendorff M, Willenbrock S, Wagner S, Schadzek P, Ngezahayo A, et al The effect of dichloroacetate in canine prostate adenocarcinomas and transitional cell carcinomas in vitro. Int J Oncol. 2016;: 2341–50. 10.3892/ijo.2016.3720 27748833

[59] Ramos-Vara JA, Miller MA, Boucher M, Roudabush A, Johnson GC. Immunohistochemical detection of uroplakin III, cytokeratin 7, and cytokeratin 20 in canine urothelial tumors. Vet Pathol. 2003; 40:55–62. 10.1354/vp.40-1-55 12627713

[60] Rasotto R, Goldschmidt MH, Castagnaro M, Carnier P, Caliari D, Zappulli V. The dog as a natural animal model for study of the mammary myoepithelial basal cell lineage and its role in mammary carcinogenesis. J Comp Pathol. 2014; 151:166–80. 10.1016/j.jcpa.2014.04.013 24975897

[61] Bonsembiante F, Benali SL, Trez D, Aresu L, Gelain ME. Histological and immunohistochemical characterization of feline renal cell carcinoma: a case series. J Vet Med Sci. 2016; 78:1039–43. 10.1292/jvms.15-0697 26888581 PMC4937140

[62] Fonseca-Alves CE, Kobayashi PE, Calderón LGR, Felisbino SL, De Carvalho Rinaldi J, Drigo SA, et al Immunohistochemical panel to characterize canine prostate carcinomas according to aberrant p63 expression. PLoS One. 2018; 13:1–16. 10.1371/journal.pone.0199173 29894516 PMC5997330

[63] Windhövel C, Harder L, Bach JP, Teske M, Grabow N, Eickner T, et al Comparison of six different silicones in vitro for application as glaucoma drainage device. Materials (Basel). 2018; 11:1–14. 10.3390/ma11030341 29495462 PMC5872920

[64] Gustafson DL, Rastatter JC, Colombo T, Long ME. Doxorubicin pharmacokinetics: Macromolecule binding, metabolism, and excretion in the context of a physiologic model. J Pharm Sci. 2002; 91:1488–501. 10.1002/jps.10161 12115848

[65] Gaver RC, George aM, Duncan GF, Morris aD, Deeb G, Faulkner HC, et al The disposition of carboplatin in the beagle dog. Cancer Chemother Pharmacol. 1988; 21:197–202. 10.1007/bf00262769 3282707

[66] Busch U, Schmid J, Heinzel G, Schmaus H, Baierl J, Huber C, et al Pharmacokinetics of meloxicam in animals and the relevance to humans. Drug Metab Dispos. 1998; 26:576–84. 9616195

[67] Chu TM, Murphy GP, Kawinski E, Mirand EA. Lncap model of human prostatic carcinoma. Cancer Res. 1983; 43:1809–18. 6831420

[68] Webber MM, Bello D, Quader S. Immortalized and tumorigenic adult human prostatic epithelial cell lines: characteristics and applications Part 2. Tumorigenic cell lines. Prostate. 1997; 30:58–64. 10.1002/(sici)1097-0045(19970101)30:1<58::aid-pros9>3.0.co;2-h 9018337

[69] Sobel R, Sadar M. Cell Lines Used in Prostate Cancer Research: a Compendium of Old and New Lines—Part 2. J Urol. 2005; 173:360–72. 10.1097/01.ju.0000149989.01263.dc 15643173

[70] Galvao JF de B, Kisseberth WC, Murahari S, Sutayatram S, Chew DJ, Inpanbutr N. Effects of gemcitabine and gemcitabine in combination with carboplatin on five canine transitional cell carcinoma cell lines. Am J Vet Res. 2012; 73:1262–72. 10.2460/ajvr.73.8.1262 22849687

[71] Budman DR, Calabro A, Kreis W. Synergistic and antagonistic combinations of drugs in human prostate cancer cell lines in vitro. Anticancer Drugs. 2002; 13:1011–6. 10.1097/00001813-200211000-00005 12439335

[72] Vallo S, Michaelis M, Wezel F, Limbart DM, Haferkamp A, Rothweiler F, et al Drug-Resistant Urothelial Cancer Cell Lines Display Diverse Sensitivity Profiles to Potential Second-Line Therapeutics. Transl Oncol. 2015; 8:210–6. 10.1016/j.tranon.2015.04.002 26055179 PMC4487788

[73] Toritsuka M, Makinodan M, Yamauchi T, Yamashita Y, Ikawa D, Komori T, et al Altered gene expression in lymphoblastoid cell lines after subculture. Vitr Cell Dev Biol—Anim. 2018; 54:523–7. 10.1007/s11626-018-0267-1 29948745

[74] Russell P, Kingsley E. Human Prostate Cancer Cell Lines. Methods Mol Med Prostate Cancer Methods Protoc. 2003; 81:21–39. 10.1385/1-59259-372-0:21 12725112

[75] Navone NM, Logothetis CJ, von Eschenbach a C, Troncoso P. Model systems of prostate cancer: uses and limitations. Cancer Metastasis Rev. 1999; 17:361–71. 10.1023/A:1006165017279 10453280

[76] Simmons JK, Elshafae SM, Keller ET, Mccauley LK, Rosol TJ. Review of Animal Models of Prostate Cancer Bone Metastasis. Vet Sci. 2014; 1:16–39. 10.3390/vetsci1010016

[77] Yang W, Soares J, Greninger P, Edelman EJ, Lightfoot H, Forbes S, et al Genomics of Drug Sensitivity in Cancer (GDSC): a resource for therapeutic biomarker discovery in cancer cells. Nucleic Acids Res. 2013; 41:D955–61. 10.1093/nar/gks1111 23180760 PMC3531057

[78] Üstün Alkan F, Bakirel T, Üstüner O, Yardibi H. In vitro effects of doxorubicin and deracoxib on oxidative-stress-related parameters in canine mammary carcinoma cells. Acta Vet Hung. 2014; 62:372–85. 10.1556/AVet.2014.012 25038953

[79] Thorn CF, Oshiro C, Marsh S, Hernandez-Boussard T, McLeod H, Klein TE, et al Doxorubicin pathways: Pharmacodynamics and adverse effects. Pharmacogenet Genomics. 2011; 21:440–6. 10.1097/FPC.0b013e32833ffb56 21048526 PMC3116111

[80] Klopfleisch R, Kohn B, Gruber AD. Mechanisms of tumour resistance against chemotherapeutic agents in veterinary oncology. Vet J. 2016; 207:63–72. 10.1016/j.tvjl.2015.06.015 26526523

[81] McMillan SK, Boria P, Moore GE, Widmer WR, Bonney PL, Knapp DW. Antitumor effects of deracoxib treatment in 26 dogs with transitional cell carcinoma of the urinary bladder. J Am Vet Med Assoc. 2011; 239:1084–9. 10.2460/javma.239.8.1084 21985349

[82] Montejo C, Barcia E, Negro S, Fernández-Carballido A. Effective antiproliferative effect of meloxicam on prostate cancer cells: Development of a new controlled release system. Int J Pharm. 2010; 387:223–9. 10.1016/j.ijpharm.2009.11.036 19963049

[83] Wolfesberger B, Walter I, Hoelzl C, Thalhammer JG, Egerbacher M. Antineoplastic effect of the cyclooxygenase inhibitor meloxicam on canine osteosarcoma cells. Res Vet Sci. 2006; 80:308–16. 10.1016/j.rvsc.2005.07.013 16182328

[84] Arenas C, Peña L, Granados-Soler JL, Pérez-Alenza MD. Adjuvant therapy for highly malignant canine mammary tumours: Cox-2 inhibitor versus chemotherapy: A case-control prospective study. Vet Rec. 2016; 179:125 10.1136/vr.103398 27377395

[85] Iturriaga MP, Paredes R, Arias JI, Torres CG. Meloxicam decreases the migration and invasion of CF41.Mg canine mammary carcinoma cells. Oncol Lett. 2017; 14:2198–206. 10.3892/ol.2017.6400 28781660 PMC5530185

[86] Yoshitake R, Saeki K, Watanabe M, Nakaoka N, Ong SM, Hanafusa M, et al Molecular investigation of the direct anti-tumour effects of nonsteroidal anti-inflammatory drugs in a panel of canine cancer cell lines. Vet J. 2017; 221:38–47. 10.1016/j.tvjl.2017.02.001 28283079

[87] Knottenbelt C, Chambers G, Gault E, Argyle DJ. The in vitro effects of piroxicam and meloxicam on canine cell lines. J Small Anim Pract. 2006; 47:14–20. 10.1111/j.1748-5827.2006.00006.x 16417605

[88] Kuczyk MA, Bokemeyer C, Serth J, Hervatin C, Oelke M, Höfner K, et al p53 overexpression as a prognostic factor for advanced stage bladder cancer. Eur J Cancer. 1995; 10.1016/0959-8049(95)00443-2 8652250

[89] Serth J, Kuczyk MA, Bokemeyer C, Hervatin C, Nafe R, Tan HK, et al P53 immunohistochemistry as an independent prognostic factor for superficial transitional cell carcinoma of the bladder. Br J Cancer. 1995; 71:201–5. 10.1038/bjc.1995.41 7819040 PMC2033435

[90] Lean FZX, Kontos S, Palmieri C. Expression of β-catenin and mesenchymal markers in canine prostatic hyperplasia and carcinoma. J Comp Pathol. 2014; 150:373–81. 10.1016/j.jcpa.2013.12.008 24529514

[91] Fonseca-Alves CE, Kobayashi PE, Rivera-Calderon LG, Laufer-Amorim R. Evidence of epithelial-mesenchymal transition in canine prostate cancer metastasis. Res Vet Sci. 2015; 100:176–81. 10.1016/j.rvsc.2015.03.001 25796368

[92] Gillet J-P, Varma S, Gottesman MM. The clinical relevance of cancer cell lines. J Natl Cancer Inst. 2013; 105:452–8. 10.1093/jnci/djt007 23434901 PMC3691946

[93] Taher L, Beck J, Liu W, Roolf C, Soller JT, Rütgen BC, et al Comparative High-Resolution Transcriptome Sequencing of Lymphoma Cell Lines and de novo Lymphomas Reveals Cell-Line-Specific Pathway Dysregulation. Sci Rep. 2018; 8:1–12. 29674676 10.1038/s41598-018-23207-7 PMC5908872

[94] Lai CL, Van Den Ham R, Van Leenders G, Van Der Lugt J, Teske E. Comparative characterization of the canine normal prostate in intact and castrated animals. Prostate. 2008; 68:498–507. 10.1002/pros.20721 18213634

[95] Akter SH, Lean FZX, Lu J, Grieco V, Palmieri C. Different Growth Patterns of Canine Prostatic Carcinoma Suggests Different Models of Tumor-Initiating Cells. 2015; 52:1027–33. 10.1177/0300985815574008 25755134

[96] Sledge DG, Patrick DJ, Fitzgerald SD, Xie Y, Kiupel M. Differences in Expression of Uroplakin III, Cytokeratin 7, and Cyclooxygenase-2 in Canine Proliferative Urothelial Lesions of the Urinary Bladder. Vet Pathol. 2015; 52:74–82. 10.1177/0300985814522819 24608632

