# Supplementary material for: Characterization of six canine prostate adenocarcinoma and three transitional cell carcinoma cell lines derived from primary tumor tissues as well as metastasis
Source: PLoS One. 2020 Mar 13;15(3):e0230272. doi: 10.1371/journal.pone.0230272 (PMC7069630; doi:10.1371/journal.pone.0230272)
Supplement: S1 Table — m = male; f = female; n = neutered; original tissue (P = Prostate, B = urinary bladder, Ln = lymph node); cell line names are explained as institution (Tiho = University of Veterinary Medicine Hannover); species (D = dog); tissue origin (Pro = prostate; Urt = urinary tract (urinary bladder)); diagnosis (Adcarc = adenocarcinoma; Carc = carcinoma; Metadcarc = metastasis of an adenocarcinoma); abbreviations of cell lines written in bold; none = cell lines have not been published yet; n.a. = tissue of patient no. 5 is missing, as the patient owners declined surgery and necropsy; *diagnosis by cytology of cells obtained by fine needle aspiration biopsy; **in total remission after a combination protocol of vincristine, asparaginase, cyclophosphamide, doxorubicin, prednisone and lomustin. (DOCX) [file pone.0230272.s001.docx]

**S1 Table: Medical patient data, tissue samples and cell lines.**

| Patient | Breed | Age [years] | Sex | Original tissue | Histological Classification | Stage and others | Cell line | Former name |
| --- | --- | --- | --- | --- | --- | --- | --- | --- |
| 1 | Briard | 10 | m | P1 | PAC | metastases (mesentery) | TihoDPro**Adcarc1258** | CT1258 [29,47,50] |
| 2 | German Rough-haired Pointer | 6 | m | P2 | PAC | intra- and paraprostatic cysts | TihoDPro**Adcarc0846** | DT08/46 [48] |
| 3 | Mixed breed | 11 | mn | P3 | PAC | metastases (lungs, brain, spinal cord, uvea) | TihoDPro**Adcarc1508** | DT15/08 [53] |
| 4 | Dobermann | 6 | mn | P4.1 | PAC | neutered due to testicular tumour, metastases (sublumbar lymph nodes) | TihoDPro**Adcarc1511.1** | none |
|  |  |  |  | Ln4.2 | PAC metastasis |  | TihoDPro**Metadcarc1511.2** | none |
|  |  |  |  | Ln4.3 |  |  | TihoDPro**Metadcarc1511.3** | none |
| 5 | Pitbull Terrier | 10 | mn | n.a. | TCC* | B-cell lymphoma**, haemangio-sarcoma | TihoDProCarc/**TCC0840** | DT08/40 [46] |
| 6 | Bolonka Zwetna | 9 | mn | P6 | TCC | tumour comprised urinary bladder and prostate, metastases (sublumbar lymph nodes) | TihoDPro**TCC1509** | DT15/09 [53] |
| 7 | Labrador Retriever | 10 | f | B7 | TCC | no metastases observed at time of surgery, metastatic (brain and other sites) and chemoresistant (cyclophosphamide) recurrence one year after surgery | TihoDUrt**TCC1506** | DT15/06 [53] |
