# Supplementary material for: Characterization of six canine prostate adenocarcinoma and three transitional cell carcinoma cell lines derived from primary tumor tissues as well as metastasis
Source: PLoS One. 2020 Mar 13;15(3):e0230272. doi: 10.1371/journal.pone.0230272 (PMC7069630; doi:10.1371/journal.pone.0230272)
Supplement: S2 Table — (DOCX) [file pone.0230272.s002.docx]

**S2 Table 2. Antibodies used for immunohistochemistry.**

| antibody | type | clone | dilution | company | | positive control (dog) | negative control |
| --- | --- | --- | --- | --- | --- | --- | --- |
| **Cellular origin** | | | | | | | |
| **pan-CK** | mouse anti-human | 34beta E12 | 1:500 | Dako, Glostrup, Denmark | | skin | mouse IgG1 |
| **CK7** | mouse anti-human | OV-TL 12/30 | 1:25 | Dako | | urinary bladder | mouse IgG1 |
| **CK8/18** | mouse anti-human | 5D3 | 1:400 | Leica biosystems, Wetzlar, Germany | | prostate | mouse IgG1 |
| **UPIII** | mouse anti-human | AU1 | 1:100 | Progen, Heidelberg, Germany | | urinary bladder | mouse IgG1 |
| **Vimentin** | mouse anti-human | V9 | 1:100 | Dako | | skin | mouse IgG1 |
| **E-Cad** | mouse anti-human | 36/E-Cad | 1:100 | BD Biosciences, Franklin Lakes, USA | | skin | mouse IgG2a |
| **Calp** | mouse anti-human | CALP | 1:1500 | Dako | | mammary gland | mouse IgG1 |
| **COX-2 and p53** | | | | | | | |
| **COX-2** | goat anti-human | polyclonal | 1:400 | Santa Cruz Biotechnology, Dallas, USA | | squamous cell carcinoma | Goat IgG1 |
| **p53** | mouse anti-human | Pab240 | 1:250 | GeneTex, Irvin, USA | | squamous cell carcinoma | mouse IgG1 |
| **Secondary antibodies** | | | | | | | |
| **for monoclonal primary antibodies** | | goat anti-mouse | | 1:200 | Vector Laboratories, Burlingame, USA | | |
| **for COX-2 primary antibodies** | | horse anti-goat | | 1:200 | Vector Laboratories, Burlingame, USA | | |
