# Supplementary figures and images for: Characterization of six canine prostate adenocarcinoma and three transitional cell carcinoma cell lines derived from primary tumor tissues as well as metastasis
Source: PLoS One. 2020 Mar 13;15(3):e0230272. doi: 10.1371/journal.pone.0230272 (PMC7069630; doi:10.1371/journal.pone.0230272)

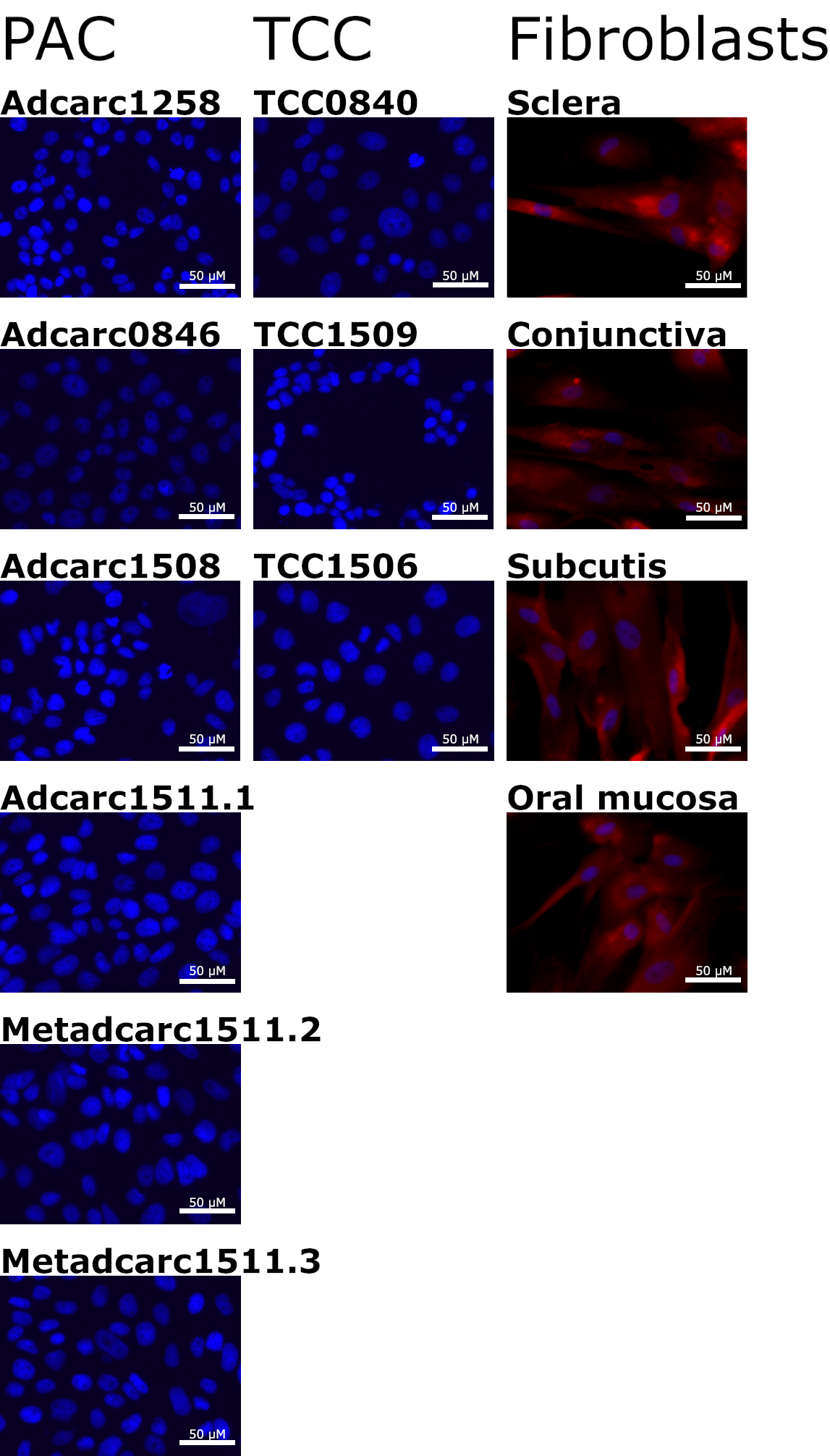

Supplement: S1 Fig — (TIFF) [file pone.0230272.s003.tiff]

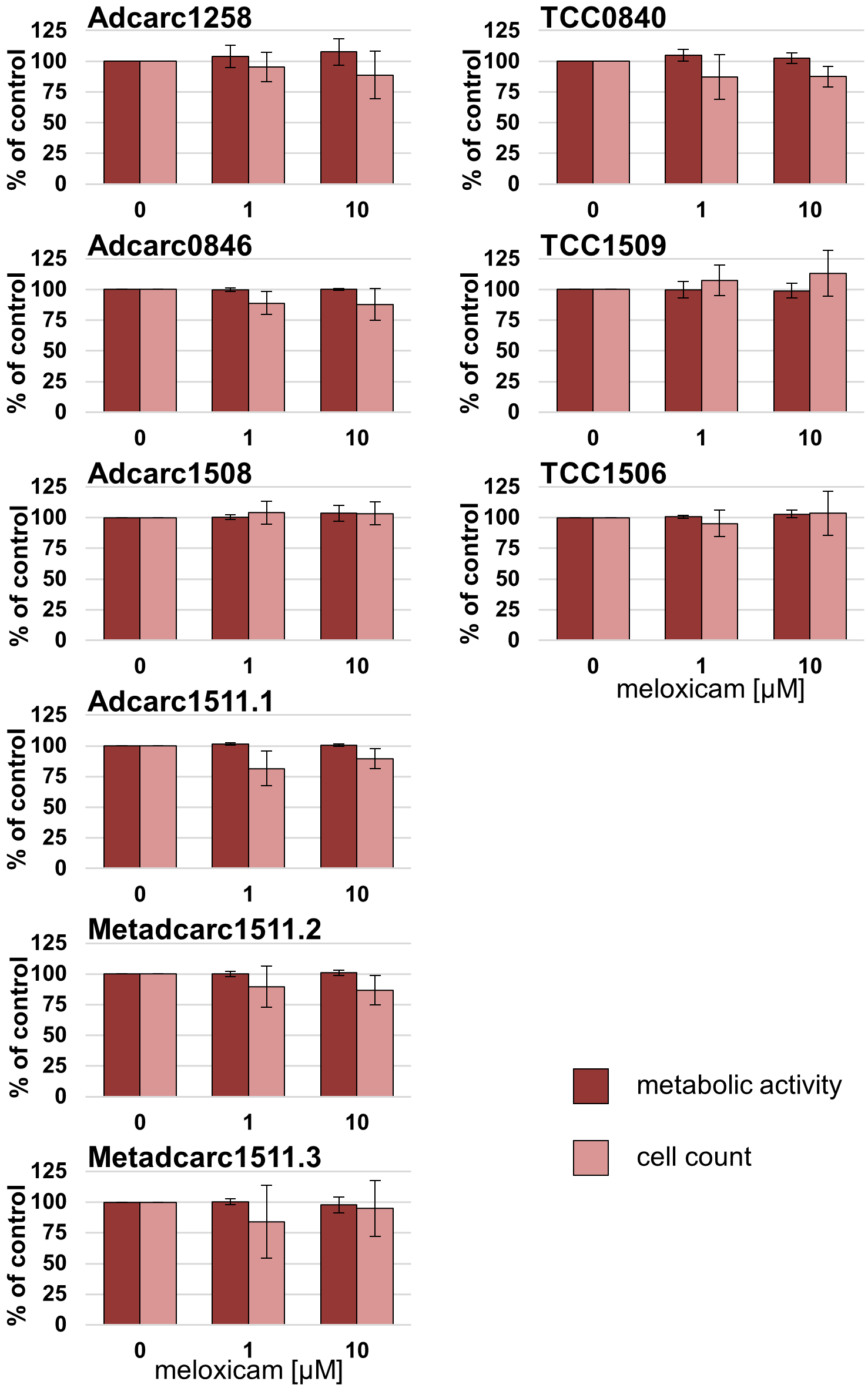

Supplement: S2 Fig — (TIF) [file pone.0230272.s004.tif]
